# Fermented Plant-Based Foods and Postbiotics for Glycemic Control—Microbial Biotransformation of Phytochemicals

**DOI:** 10.3390/molecules31020360

**Published:** 2026-01-20

**Authors:** Emilia Cevallos-Fernández, Elena Beltrán-Sinchiguano, Belén Jácome, Tatiana Quintana, Nadya Rivera

**Affiliations:** Centro de Investigación de Alimentos (CIAL), Facultad de Ciencias de la Ingeniería e Industrias, Universidad UTE, Quito 170129, Ecuador; mariab.jacome@ute.edu.ec (B.J.); tatiana.quintana@ute.edu.ec (T.Q.); nrivera@ute.edu.ec (N.R.)

**Keywords:** plant-based fermented foods, postbiotics, glycemic control, EPS, sourdough, kombucha, natto, gut–liver axis, γ-PGA, zero hunger, good health and well-being, responsible consumption and production

## Abstract

Plant-based fermented foods are increasingly promoted for glycemic control, yet their mechanisms and clinical impact remain incompletely defined. This narrative review synthesizes mechanistic, preclinical, and human data for key matrices—kimchi and other fermented vegetables, tempeh/miso/natto, and related legume ferments, kombucha and fermented teas, plant-based kefir, and cereal/pulse sourdoughs. Across these systems, microbial β-glucosidases, esterases, tannases, and phenolic-acid decarboxylases remodel polyphenols toward more bioaccessible aglycones and phenolic acids, while lactic and acetic fermentations generate organic acids, exopolysaccharides, bacterial cellulose, γ-polyglutamic acid, γ-aminobutyric acid, and bioactive peptides. We map these postbiotic signatures onto proximal mechanisms—α-amylase/α-glucosidase inhibition, viscosity-driven slowing of starch digestion, gastric emptying and incretin signaling, intestinal-barrier reinforcement, and microbiota-dependent short-chain–fatty-acid and bile-acid pathways—and their downstream effects on AMPK/Nrf2 signaling and the gut–liver axis. Animal models consistently show improved glucose tolerance, insulin sensitivity, and hepatic steatosis under fermented vs. non-fermented diets. In humans, however, glycemic effects are modest and highly context-dependent: The most robust signal is early postprandial attenuation with γ-PGA-rich natto, strongly acidified or low-glycemic sourdough breads, and selected kombucha formulations, particularly in individuals with impaired glucose regulation. We identify major sources of heterogeneity (starters, process parameters, substrates, background diet) and safety considerations (sodium, ethanol, gastrointestinal symptoms) and propose minimum reporting standards and trial designs integrating metabolomics, microbiome, and host-omics. Overall, plant-based ferments appear best positioned as adjuncts within cardiometabolic dietary patterns and as candidates for “purpose-built” postbiotic products targeting early glycemic excursions and broader metabolic risk.

## 1. Introduction

Plant-based fermented foods sit at the intersection of two converging themes in cardiometabolic research: the central role of postprandial glycemia in type 2 diabetes (T2D) and cardiovascular risk and the emerging view of diet–microbiome interactions as a determinant of inter-individual metabolic responses. Meta-omics syntheses now link habitual diet, gut microbiome structure, and microbe-derived metabolites to insulin resistance, dyslipidemia, and cardiometabolic endpoints [1,2]. At the same time, large-scale personalized-nutrition studies show that glycemic excursions after ostensibly similar carbohydrate loads vary widely between individuals, with models that incorporate microbiome features, lifestyle, and clinical variables outperforming static food-based indices [3,4,5]. These observations motivate closer scrutiny of “complex” foods that deliver both carbohydrates and microbiome-active metabolites, including fermented plant products.

Microbial fermentation—deliberate growth and metabolism of food-associated microbes—remodels plant matrices in ways that go beyond simple preservation. It alters texture and flavor, increases shelf life, and generates a repertoire of small molecules and polymers that are scarce or absent in the starting substrate [6,7]. The International Scientific Association for Probiotics and Prebiotics (ISAPP) now distinguishes fermented foods from probiotics and defines postbiotics as “preparations of inanimate microorganisms and/or their components that confer a health benefit,” framing fermented foods as complex vehicles of live cells, dead cells, and microbe-derived metabolites [8,9]. Recent conceptual work further introduces the idea of an “extended microbiome,” in which metabolites originating from food fermentations function as human-relevant microbial signals—bridging environmental microbiomes, food processing, and host biology [10,11]. Within this framework, the chemical complexity of fermented foods is not a nuisance but a mechanistic opportunity.

Plant-based fermented foods are particularly salient because they are widely consumed across cultures (e.g., kimchi and other fermented vegetables, miso/natto/tempeh and other soybean ferments, kombucha and fermented teas, water kefir, and cereal and pulse sourdoughs), fit within plant-forward dietary patterns, and often replace or complement higher-glycemic staples [12,13,14,15,16]. Contemporary reviews and scoping syntheses document rich microbial consortia and metabolite formation in these systems—from mannitol, organic acids, γ-aminobutyric acid (GABA), and exopolysaccharide (EPS) in kimchi and sauerkraut to isoflavone aglycones and bioactive peptides in fermented soy, bacterial cellulose (BC) and remodeled polyphenols in kombucha, and dextran-rich grains in water kefir [17,18,19,20]. Parallel clinical and preclinical work suggests that some of these foods can improve glycemic or broader metabolic markers: Trials and meta-analyses report modest improvements in fasting glucose or Homeostasis Model Assessment of Insulin Resistance (HOMA-IR) with kefir, fermented fruits/vegetables, or mixed fermented-food interventions; mechanistic and cohort data highlight potential benefits of kimchi, miso, natto, kombucha, kefir, and sourdough on lipids, adiposity, liver steatosis, and functional gastrointestinal outcomes [21,22,23,24]. However, the magnitude and direction of glycemic effects are heterogeneous, and in many trials, glycemic outcomes are neutral despite improvements in other cardiometabolic or gut-related endpoints [23,25].

Mechanistic studies offer several plausible routes by which fermented plant matrices could influence early postprandial glycemia. Fermentation-driven enzymatic remodeling increases the proportion of aglycone polyphenols and liberates smaller phenolic acids, which can inhibit α-amylase and α-glucosidase and modulate incretin signaling [6,26,27]. Organic acids (lactate, acetate, gluconate, citrate) and delivered short-chain fatty acids (SCFAs) can attenuate early glucose excursions in some contexts, alter gastric emptying, and engage nutrient-sensing G protein–coupled receptors on enteroendocrine cells [28,29]. Fermentation-associated polymers—EPS from lactic acid bacteria (LAB), bacterial cellulose in kombucha, and γ-polyglutamic acid (γ-PGA) in natto—build viscosity and viscoelastic networks that can slow starch hydrolysis and glucose diffusion, as illustrated by crossover meal tests with γ-PGA-rich natto and by detailed rheological work on EPS and BC systems [30,31]. Additional pathways include intestinal-barrier reinforcement with reduced endotoxemia and liver inflammation, modulation of SCFA and bile-acid signaling via free fatty acid receptors 2 and 3 (FFAR2/3) and Farnesoid X receptor (FXR)/Takeda G protein-coupled receptor 5 (TGR5), and dipeptidyl peptidase-IV (DPP-IV-inhibitory) or Angiotensin-Converting Enzyme-Inhibitory (ACE-inhibitory) peptides generated during legume and cereal fermentations [32,33,34].

Yet, current evidence rarely connects these mechanistic layers within the same study. Many trials report only pH or titratable acidity without quantifying organic acids, residual sugars, ethanol, EPS, or γ-PGA, or key phenolic/peptide markers in the products actually consumed [10,35]. Host-side measurements often stop at fasting glucose and lipids, with limited use of standardized postprandial sampling, gut hormones, SCFAs, bile acids, or multi-omics [1,10]. As a result, it is difficult to ascribe observed glycemic effects (or null findings) to specific fermentation-derived metabolites, matrix properties, or host pathways. Recent consensus and methodological papers explicitly call for more rigorous identification and quantification of fermented-food exposures and for analytical frameworks that link matrix chemistry to clinical endpoints [24,25].

Inter-individual variability further complicates interpretation. Continuous glucose monitoring studies indicate that glycemic responses to bread and other carbohydrate foods are strongly person-specific and associated with microbiome and clinical features, implying that any glycemic modulation by fermented plant foods will likely be stratified by host phenotype [3,36]. Against this backdrop, existing syntheses on fermented foods, sourdough, kefir, and botanical ferments typically treat “fermented” as a broad exposure and focus on metabolic syndrome, T2D, satiety, or gastrointestinal health, rather than dissecting the plant-based subgroup with attention to postbiotic chemistry, polymer physics, and enzyme-to-metabolite-to-endpoint attribution [3,24,36,37].

Therefore, there is a need for an integrative, matrix-anchored synthesis that focuses specifically on plant-based fermented foods and glycemic control and that treats fermented-food metabolites and polymers as measurable exposures rather than black boxes. The objective of this article is to provide such a structured narrative review by (i) mapping the main plant-based fermented matrices relevant to glycemic control and their characteristic microbial consortia and metabolites; (ii) identifying postbiotic metabolites and polymer features most likely to blunt early postprandial glycemia; (iii) synthesizing evidence from in vitro models, animal studies, and human interventions—including head-to-head comparisons with non-fermented controls; and (iv) proposing analytical anchors and minimal reporting items needed to connect enzyme-level mechanisms and matrix chemistry to clinical glycemic endpoints and to inform the design of purpose-built plant ferments and postbiotic preparations for metabolic health. 

## 2. Methods—Evidence Identification and Synthesis

### 2.1. Review Type, Scope, and Rationale

We conducted a structured narrative review with scoping elements focused on plant-based fermented foods and glycemic control. A fully registered systematic review with meta-analysis was not adopted for three main reasons. First, our primary aim was integrative and mechanistic—to link food matrix chemistry (polymers, organic acids, phenolic remodeling, peptides) with proximal mechanisms (enzyme inhibition, gastric emptying/incretins, microbiota-derived metabolites) and glycemic endpoints across diverse plant-based ferments. This required incorporating heterogeneous evidence streams, including in vitro experiments, animal models, human meal tests, longer interventions, and analytical/methodological studies that often do not conform to a single PICO framework or share harmonized outcome measures.

Second, the underlying evidence base is highly heterogeneous in terms of exposures (different fermented foods, starters, substrates, co-ingredients, fermentation conditions), outcome metrics (glycemic indices, incremental areas under the curve, surrogate markers), and analytical reporting (e.g., presence/absence of organic-acid profiling, polymer rheology, or detailed metabolomics). This heterogeneity frequently precludes meaningful quantitative pooling and limits the applicability of conventional risk-of-bias tools that are optimized for more uniform clinical trial datasets. Instead, a structured narrative approach allowed us to group studies by matrix type and mechanistic pathway and to summarize direction and approximate magnitude of effects while explicitly highlighting gaps and inconsistencies.

Third, because many key contributions are analytical or mechanistic (e.g., characterization of exopolysaccharides, bacterial cellulose, γ-PGA, or polyphenol biotransformation) without direct glycemic endpoints, a scoping component was needed to map this broader landscape and decide which mechanistic data are sufficiently relevant to include alongside clinical findings.

To mitigate the risk of selection bias inherent in narrative syntheses, we (i) pre-specified research questions (Section 2.2), plant-based food categories, and mechanistic domains of interest; (ii) used explicit eligibility criteria and targeted search blocks combining foods, mechanisms, and analytical terms in Scopus and PubMed (Section 2.3); (iii) applied a two-stage screening process (titles/abstracts followed by full text) against these criteria; and (iv) complemented database searches with backward and forward citation chasing from key articles to capture additional relevant studies. Within the included body of evidence, we deliberately reported both positive and null findings and weighted interpretation by study design, sample size, and analytical transparency, as detailed in the Section 2.7, Section 2.8 and Section 2.12. In this sense, the present work is best viewed as a matrix-anchored, mechanistically oriented structured narrative/scoping synthesis that adopts several principles of systematic reviewing (transparent search, explicit eligibility, structured extraction), while acknowledging that the heterogeneity of the field does not yet support a formal meta-analysis or comprehensive risk-of-bias grading.

### 2.2. Research Questions

1. Which postbiotic metabolites and polymer features of plant-based fermented foods are most likely to blunt early postprandial glycemia?
2. What analytical anchors and minimal reporting items enable reproducible attribution from mechanisms to clinical endpoints?

### 2.3. Information Sources and Search Strategy

We searched Scopus and PubMed iteratively through November 2025. For each section and subsection, we pre-specified topics/subtopics and keyword blocks (foods × mechanism × analytics), then refined terms as signal accumulated. Example blocks included:

- (kombucha OR kefir OR kimchi OR sourdough) AND (“organic acid*” OR “residual sugar*” OR ethanol) AND (HPLC OR GC) AND (report* OR validation).
- (exopolysaccharide* OR dextran OR “bacterial cellulose”) AND (rheology* OR viscosity OR “degree of substitution”) AND (kefir OR kombucha OR nata).
- (isoflavone* OR genistein OR daidzein) AND (LC-MS OR UPLC-MS/MS OR MRM) AND (soy milk OR plant milk) AND (quantification OR validation).

### 2.4. Eligibility Criteria

- Inclusion. Peer-reviewed studies on plant-based fermented foods (vegetables/kimchi/sauerkraut; soy/tempeh/miso/natto; tea/kombucha; plant-based kefirs; cereal/pulse sourdoughs) that reported at least one of (i) glycemic endpoints (e.g., postprandial glucose/insulin incremental area under the curve (iAUC)), (ii) proximal mechanisms (α-amylase/α-glucosidase inhibition, gastric emptying proxies, incretins), (iii) matrix attributes relevant to transport (EPS/bacterial cellulose (BC)/γ-PGA yield, molecular weight (MW), rheology), or (iv) targeted/untargeted metabolite analytics (organic acids, residual sugars/ethanol, phenolics/isoflavones, SCFAs). Human studies were prioritized; rodent/in vitro papers were included when they clarified mechanisms or matrix analytics directly relevant to human translation.
- Exclusion. Non-fermented comparators without a fermented arm; dairy-only kefir unless used as a mechanistic benchmark; editorials, non-peer-reviewed sources; studies lacking primary data on outcomes of interest.

### 2.5. Study Selection

A two-stage screening (titles/abstracts → full text) was applied to all records retrieved, with deduplication before screening. When relevancy was uncertain at abstract level, the record was conservatively retained for full-text review. We also used citation chasing (backward/forward) from key papers to capture additional eligible studies.

### 2.6. Data Extraction

From each included item we extracted the following information: study design; sample size/population (or matrix/model); fermented product identity and dose; fermentation parameters (substrate/tea, time–temperature, pH/titratable acid; when available: EPS/BC/γ-PGA content, MW proxies, rheology); comparators; outcomes (postprandial glucose/insulin iAUC, incretins, enzyme assays, gastric emptying proxies); analytical methods (High-Performance Liquid Chromatography (HPLC)/Gas Chromatography–Mass Spectrometry (GC)/Liquid Chromatography–Mass Spectrometry (LC–MS)) settings, derivatization, internal standards, Limit of Detection (LOD)/Limit of Quantification (LOQ); and main quantitative results.

### 2.7. Synthesis Approach

Given the marked heterogeneity in fermented foods, analytical characterization, and reported endpoints, we performed a structured narrative synthesis rather than a quantitative meta-analysis. The synthesis was organized along three intersecting axes: (i) matrix category (fermented vegetables; soy/legume ferments; cereal/pulse and sourdough products; fermented beverages), (ii) level of evidence (in vitro and analytical studies; animal models; acute human postprandial tests; intermediate and longer-term human interventions), and (iii) mechanistic pathway (enzyme inhibition, gastric emptying and incretin signaling, intestinal barrier and gut–liver axis, microbiota-derived SCFA and bile-acid signaling, and cross-cutting cellular pathways such as AMPK/Nrf2).

Within each matrix category, human randomized controlled trials and crossover studies were presented first, followed by other human interventions (e.g., pre–post designs, pilot trials), and then animal and in vitro models that contributed mechanistic and matrix-chemistry insight. Acute postprandial studies were explicitly separated from multi-week or longer interventions to distinguish short-term modulation of glucose excursions from adaptations in insulin sensitivity, body weight, or lipid metabolism.

Where possible, we extracted or recalculated effect estimates (e.g., absolute and percentage changes in postprandial glucose or insulin iAUC, fasting glycemia, HOMA-IR, incretins) and reported them descriptively rather than pooling them statistically. Mechanistic outcomes (SCFAs, bile acids, incretins, intestinal permeability markers, inflammatory cytokines, signaling proteins such as AMPK/Nrf2) were interpreted in the context of the reported matrix properties (organic acid profile, residual sugars/ethanol, polymer content and rheology, phenolic or isoflavone remodeling). Throughout the Results, we deliberately juxtaposed positive and neutral findings, highlighted plausible sources of heterogeneity (differences in fermentation conditions, substrates, co-ingredients, background diet, and metabolic status), and clearly indicated when a proposed mechanism was supported predominantly by preclinical data versus human evidence.

### 2.8. Quality and Certainty Considerations

This work was not a registered systematic review, and no formal risk-of-bias or GRADE-style certainty assessment was applied. Instead, we used a qualitative weighting framework to guide the emphasis of our synthesis and the strength of inferences. Priority was given to adequately powered human randomized controlled or crossover trials with appropriate comparators, standardized test meals, and transparent reporting of primary and secondary endpoints (including variance measures). Shorter-term, uncontrolled human studies and observational data were treated as supportive or hypothesis-generating. Animal and in vitro models were primarily used to elucidate mechanisms or matrix chemistry and are interpreted as such rather than as direct evidence of clinical efficacy.

When appraising individual studies, we considered randomization, blinding, run-in and washout periods, completeness of outcome reporting, and internal consistency of results as pragmatic indicators of methodological quality. Particular weight was given to studies that provided detailed compositional characterization of the fermented matrices (e.g., pH/titratable acidity, organic acids, residual sugars/ethanol, polymer yields, molecular-weight proxies, postbiotic metabolite profiling with validated HPLC/GC/LC–MS methods), because these data are essential for attributing glycemic effects to specific matrix features. Conversely, when matrix chemistry was poorly described, we treated mechanistic conclusions as provisional.

For cross-cutting pathways such as AMPK/Nrf2 or bile-acid signaling via FXR/TGR5, we explicitly distinguish in the Results and Discussion between mechanisms supported by human evidence and those that remain largely preclinical. Across all food categories, we sought to reduce publication and confirmation bias by actively including neutral or negative trials when identified and by framing the overall certainty of evidence as modest and context-dependent, pending larger, longer, and better-characterized human studies.

### 2.9. Reporting and Transparency

Reporting was informed by PRISMA and PRISMA-ScR principles (clear eligibility criteria, description of search strategies, selection logic, reasons for inclusion/exclusion, and predefined data items), but the present work should be considered a structured narrative/scoping-style synthesis rather than a registered systematic review. We provide an overview of the search process and selection in the Methods and transparently indicate where evidence is predominantly preclinical, where human data are available, and where findings are neutral or inconsistent.

### 2.10. Role of Targeted DOI Sets

For Section 4.2.4 (polymer viscosity and rheology) and Section 4.2.6 (analytical anchors), we incorporated targeted DOI sets curated a priori to cover rheology/polymer physics and LC–MS/GC method validation. These studies were included even when they lacked glycemic endpoints, specifically to bridge matrix properties (e.g., exopolysaccharides, bacterial cellulose, γ-PGA, phenolic and isoflavone profiling) to mechanistic pathways and, where possible, to clinical outcomes. Their role is explicitly mechanistic and methodological, complementing rather than substituting human intervention data.

### 2.11. Organization of Evidence Synthesis

Beyond matrix chemistry (Section 3 and Section 4), the narrative synthesis is organized around four mechanistic and translational domains: Section 5 (mechanistic pathways linking fermented foods to glycemic control: intestinal barrier/gut–liver axis; incretin signaling and DPP-IV; AMP-activated protein kinase (AMPK)/Nuclear factor erythroid 2-related factor 2 (Nrf2) interfaces; microbiota crosstalk via SCFA and bile-acid receptors), Section 6 (evidence from preclinical models and human interventions, including head-to-head comparisons and safety signals), Section 7 (sources of heterogeneity and methodological gaps), and Section 8 (recommendations and study-design guidance). Throughout these sections, we distinguish mechanisms supported primarily by human data from those grounded mainly in animal or in vitro models and emphasize how matrix characterization conditions the interpretation of glycemic outcomes.

### 2.12. Limitations

This is a structured narrative/scoping synthesis—not a registered systematic review—and we did not apply formal risk-of-bias or certainty grading. Searches were targeted (PubMed/Scopus) and may have missed studies outside our keyword blocks or databases. Marked heterogeneity in product chemistry (e.g., residual sugars/organic acids, EPS/BC/γ-PGA and rheology), dose and serving context, background diet, participant phenotypes, and sampling windows limits comparability and precluded meta-analysis. Analytical non-uniformity (inconsistent validation, internal standards, and pre-analytical handling for SCFAs, phenolics, and other metabolites) weakens direct attribution along the enzyme → metabolite → endpoint chain. Many human data derive from small, short-term crossovers in healthy participants, reducing generalizability; selective reporting and industry involvement may also introduce bias. We sought to mitigate these constraints by prioritizing human RCTs/crossovers when available, foregrounding matrix analytics, aligning postprandial time-courses, deliberately including neutral findings, and proposing standardized reporting items (Table 1 and Table 2) to support higher-certainty evidence in future work.

## 3. Plant-Based Foods: Scope and Relevance

Fermentation is the purposeful microbial and enzymatic remodeling of plant matrices that reconfigures the chemical and physical context of the food, often enhancing bioaccessibility and generating molecules that are absent or scarce in the raw substrate (e.g., organic acids, small phenolics, bioactive peptides, microbial polymers, and cell-wall-derived structures) [7]. In plant systems—spanning vegetables, soy, and other legumes, cereals, and teas—this remodeling creates a diverse repertoire of small molecules and cell-derived components with potential metabolic relevance, as highlighted in recent syntheses linking food fermentation to host physiology and metabolic health [11].

In this section, we map the main plant-based fermented food categories most germane to glycemic control—fermented vegetables (e.g., kimchi, sauerkraut, pickled teas); soy and legume ferments (e.g., tempeh, miso, natto, doenjang); cereal- and pulse-based sourdough and related products (e.g., breads, pasta, biscuits); and fermented beverages (e.g., kombucha, water kefir). For each, we summarize predominant microbial consortia and characteristic bioactives, highlighting how fermented matrices differ from raw or cooked counterparts in terms of acidity, residual sugars, phytochemical profiles, viscosity-building polymers, and microbially derived metabolites.

Because this review covers both foods containing live microorganisms and preparations where the biological activity is largely attributable to inanimate microbial cells or their components, we explicitly distinguish, where possible, between effects of the whole fermented matrix and effects that can be ascribed to postbiotics (in line with the ISAPP definition). The specific biochemical transformations, the formation of postbiotics, and mechanistic routes through which fermented plant foods may influence glycemic control are developed in Section 4, Section 5 and Section 6.

A synoptic overview of plant-based fermented matrices, their predominant microbes, key biotransformed phytochemicals, characteristic postbiotic metabolites, and minimum standardization fields (e.g., pH/titratable acidity, residual sugars/ethanol, organic-acid profile, polymer metrics) is provided in Table S1.

### 3.1. Key Plant-Based Fermented Foods Relevant to Glycemic Control

#### 3.1.1. Kimchi and Other Fermented Vegetables

Kimchi is a lactic fermentation of Brassica vegetables in which heterofermentative *Leuconostoc* species typically dominate early and are followed by lactobacilli; these consortia commonly generate mannitol (via fructose reduction), lactic and acetic acids, EPS, and a range of peptides that reshape the chemical context of the vegetable matrix [12,17]. Contemporary overviews of kimchi microbiology and metabolite formation consistently highlight these endpoints—especially mannitol and organic acids—as hallmarks of plant-based lactic fermentations [13].

As a recent example, a serial co-fermentation using *Leuconostoc citreum* S5 and *Lactiplantibacillus plantarum* KS2020 yielded a GABA-enhanced kimchi beverage, with viable counts reaching approximately 9.11–9.42 log colony-forming unit (CFU)/mL on day 1 at 5% sucrose; mannitol synthesized by *Leuconostoc* was subsequently depleted as *L. plantarum* grew, illustrating strain-level cross-feeding within kimchi fermentations [51]. While GABA enrichment is analytically robust and often highlighted as a potential antidiabetic lever, direct evidence that kimchi-derived GABA per se improves glycemic control in humans is scarce and derives mainly from preclinical models or studies with isolated GABA rather than from kimchi intervention trials. In practice, organic acids, mannitol, and EPS produced during vegetable lactic fermentation have clearer proximate links to gastric emptying, enzyme inhibition, osmotic effects, and barrier/inflammatory signaling, and are more likely to underlie the modest but measurable glycemic and metabolic changes observed in kimchi studies described below.

Sauerkraut—a spontaneous lactic fermentation of cabbage—shares core LAB consortia with kimchi (for example, *Leuconostoc* and *Lactiplantibacillus/Pediococcus*) and accumulates lactic and acetic acids together with strain-dependent EPS and volatile esters. In artisanal sauerkraut from two producers, pH fell rapidly within the first week (from 5.7 ± 0.2 to 3.8 ± 0.1 in SK1; from 5.7 ± 0.3 to 4.0 ± 0.02 in SK2), while LAB counts rose from approximately 3.4–3.6 to approximately 5.7–8.1 log CFU/mL by day 7 and reached 6.4 ± 0.5 (SK1) and 8.2 ± 0.2 (SK2) log CFU/mL by day 35; community succession shifted from early *Leuconostoc mesenteroides/pseudomesenteroides* to later *L. plantarum/paraplantarum*, with 220 LAB isolates recovered overall. Proton Nuclear Magnetic Resonance (^1^H-NMR) profiling highlighted lactic/acetic acids and small-molecule signatures, and brine exposure modulated cytokine responses (↑Tumor necrosis factor-alpha (TNF-α)/Interleukin (IL)-6 with ↑IL-10 at 24 h) in a Peripheral Blood Mononuclear Cells (PBMC)–Caco-2 co-culture, consistent with postbiotic-driven immunomodulation [52,53].

Clinically, in a dormitory-controlled randomized feeding trial, 100 healthy adults were assigned to low (15 g/day) or high (210 g/day) kimchi intake for seven days while receiving identical menus and maintaining habitual physical activity. Both groups showed significant decreases in fasting blood glucose, total cholesterol, and low-density lipoprotein (LDL)-cholesterol after seven days, with dose-dependent effects. Lipid-lowering responses were more pronounced among participants with baseline total cholesterol ≥ 190 mg/dL or LDL-cholesterol ≥ 130 mg/dL. The fall in fasting blood glucose was significantly greater with high vs. low intake (between-group *p* = 0.003), with mean changes of roughly −5.3 mg/dL and −2.0 mg/dL, respectively [54]. These changes are modest in absolute magnitude but suggest that fermented vegetables can favorably modulate short-term cardiometabolic risk markers under controlled dietary conditions.

In prediabetes, a randomized 8-week crossover feeding study (*n* = 21) compared fresh (1-day) vs. fermented (10-day) kimchi, separated by a 4-week washout. Both periods reduced body weight, body mass index, and waist circumference, but fermented kimchi additionally decreased insulin resistance and increased insulin sensitivity, with significant improvements in HOMA-IR and Quantitative Insulin Sensitivity Check Index (QUICKI) (reported *p* = 0.004 and *p* = 0.028, respectively). The proportion of participants with improved glucose tolerance was 33.3% during fermented-kimchi intake vs. 9.5% with fresh kimchi; systolic and diastolic blood pressure (BP) also fell during the fermented period [21].

Mechanistically, strains isolated from kimchi can attenuate starch digestion in vitro: *L. plantarum* LRCC5314 inhibited α-amylase and α-glucosidase activities by approximately 72.9% and approximately 51.2%, respectively, supporting a plausible pathway for blunting early postprandial glycemic excursions [55]. In overweight and obese adults, a randomized crossover study (*n* = 22; two 4-week periods separated by a 2-week washout) reported greater net reductions with fermented compared with fresh kimchi in fasting glucose, total cholesterol, percent body fat, and systolic and diastolic BP; fasting insulin also tended to decline after fermented kimchi [56].

Beyond kimchi, observational data on other fermented vegetables point in a similar direction. In a prospective cohort from China (*n* = 6640 analyzed; approximately 10-year follow-up), regular consumption of pickled vegetables was associated with a lower risk of incident diabetes in a dose-responsive fashion (0–0.5 kg/month: odds ratio (OR) 0.77, 95% confidence interval (CI) 0.63–0.94; >0.5 kg/month: OR 0.37, 95% CI 0.23–0.60), and fermented bean curd intake was likewise associated with reduced risk (OR 0.68, 95% CI 0.55–0.84) after multivariable adjustment [57]. Short-term high vs. low kimchi intake has also been linked with shifts in gut microbiota composition, including reductions in potentially pathogenic taxa and significant changes across dozens of species in the high-intake group [58].

Overall, kimchi exemplifies how plant-based lactic fermentation can yield metabolite signatures (mannitol, organic acids, EPS, bioactive peptides, and other postbiotic components) with mechanistic links to glycemic control and measurable signals in humans—from short-term reductions in fasting glycemia and atherogenic lipids in healthy adults to improved insulin sensitivity and glucose tolerance in prediabetes. At the same time, recipe composition (notably salt and co-ingredients), fermentation length, and starter strain selection vary widely and likely contribute to between-study heterogeneity and to the modest, context-dependent magnitude of effects, an issue revisited in Section 7.

#### 3.1.2. Tempeh, Miso, and Soy-Based Ferments

Fermenting soy increases β-glucosidase activity and converts isoflavone glycosides into aglycones, while proteolysis liberates bioactive peptides—changes that can plausibly affect glycemic handling via intestinal incretin and systemic signaling pathways. Comparable fermentation-driven peptide release has also been reported in emerging fermented legume products beyond soy, with enrichment of short, bioactive sequences (including DPP-IV-inhibitory peptides) in chickpea-, lentil-, and mixed-legume matrices following controlled fermentation and hydrolysis [59]. These developments suggest that the glycemic-relevant mechanisms described for traditional soy ferments may extend to a broader class of plant-based fermented protein foods.

In humans, an open-label, parallel intervention in healthy women (*n* = 16) included a 7-day soy washout followed by 28 days of either steamed tempeh (100 g/day; *n* = 10) or soymilk (200 mL/day; *n* = 6). Fecal Quantitative Polymerase Chain Reaction (qPCR) showed significant microbiota shifts with tempeh: *Akkermansia muciniphila* increased approximately 37-fold by day 29 and *Bifidobacterium* rose approximately 1.5-fold, whereas both taxa decreased in the soymilk group. Although glycemic endpoints were not measured, the magnitude and direction of these changes are consistent with a gut-mediated route through which soy fermentation might influence glucose homeostasis [60].

Beyond microbiological remodeling, modified tempeh formulations have been tested for glycemic and microbiome endpoints in diabetic rodent models. In streptozotocin–nicotinamide diabetic rats fed for 4 weeks with diets where casein was partially replaced by standard tempeh (15% or 30% of protein) or by a lactic-acid-bacteria-acidified “modified tempeh” (15% or 30%), groups differed significantly in fasting serum glucose (*p* < 0.001). The modified product also altered the cecal short-chain fatty acid profile (acetate/propionate/butyrate, all *p* < 0.001), increased bacterial diversity, and shifted the Firmicutes/Bacteroidetes ratio upward relative to diabetic controls; compositionally, the modified tempeh had higher protein (19.85 ± 0.02% vs. 17.03 ± 0.02), fat (10.85 ± 0.06% vs. 9.27 ± 0.01), and dietary fiber (8.20 ± 0.19% vs. 5.92 ± 0.02) than regular tempeh, indicating a distinct nutrient and postbiotic matrix relevant to host metabolism [61].

A companion 30-day study using the same processing strategy reported that the modified tempeh contained a higher LAB load (9.99 ± 0.09 vs. 7.74 ± 0.07 log CFU; *p* < 0.001) and improved glycemic readouts, with significant pre–post differences in serum glucose and insulin overall and strong negative correlations between gut-microbiota diversity and both Δ glucose (r = −0.63; *p* < 0.001) and Δ insulin-resistance index (r = −0.54; *p* = 0.003), supporting a microbiome-linked route to glycemic control in this preclinical model [62].

For miso, population data in outpatients with T2D indicate sex-specific associations with glycemic control. Habitual miso consumption correlated with lower mean hemoglobin A1c (HbA1c) and reduced visit-to-visit variability among women after multivariable adjustment (e.g., β for average HbA1c approximately −0.25, *p* = 0.009; similar directions for standard deviation (SD) and coefficient of variation (CV)), with no association in men [63]. These findings suggest that, at least in women with T2D, fermented soy pastes may contribute modestly to improved glycemic stability within the broader dietary pattern.

In acute settings, a randomized crossover single-meal study in healthy adults testing a legume-based miso-type sauce did not alter postprandial glucose or insulin vs. control, despite increases in plasma antioxidant capacity and favorable short-term lipid signals—underscoring that compositional or antioxidant improvements in fermented products do not automatically translate into detectable acute glycemic effects in low-risk populations [64].

In a high-fat/high-sucrose mouse model, dietary miso suppressed diet-induced impairments in glucose handling: Both intraperitoneal glucose tolerance (iPGTT) and insulin tolerance (ITT) area-under-the-curve values were significantly lower vs. control (*n* = 6/group; *p* < 0.05). Miso feeding also improved muscle function (higher grip strength and soleus muscle mass) and reduced muscle inflammation, with downregulation of Tumor Necrosis Factor alpha (TNF-α) and Chemokine (C–C motif) ligand 2 (Ccl2) expression (*p* < 0.05). SCFAs increased across matrices—including feces, serum, skeletal muscle, and epididymal white adipose tissue—supporting a gut–muscle axis contribution to metabolic effects, although these remain preclinical findings [65].

In the same outpatient framework, a cross-sectional analysis (*n* = 300) stratified participants by urine-estimated salt intake and habitual miso consumption. Obesity prevalence was 77.8% in high-salt/no-miso (+/−), 40.2% in high-salt/with-miso (+/+), 26.0% in low-salt/with-miso (−/+), and 34.8% in low-salt/no-miso (−/−). Using the high-salt/no-miso group as reference, adjusted odds ratios were 0.07 (95% CI 0.02–0.26; *p* < 0.001) for (−/+), 0.14 (0.04–0.51; *p* = 0.003) for (+/+), and 0.16 (0.03–0.76; *p* = 0.022) for (−/−), suggesting that habitual miso consumption attenuated the salt–obesity association in this cohort [66]. While these data are observational and not specific to glycemia, they are consistent with broader metabolic benefits of fermented soy pastes in real-world dietary contexts.

Beyond microbiota signals, natto—a *Bacillus*-fermented soy food rich in viscous γ-PGA—has shown acute glycemic effects in randomized crossover meal tests. In healthy adults, a three-arm trial comparing white rice, low-γ-PGA natto, and high-γ-PGA natto found that the glucose IAUC_0–120_ was approximately 20.1% lower with high-γ-PGA natto vs. white rice (*p* < 0.05), with early-phase (0–45 min) suppression vs. low-γ-PGA natto as well; insulin IAUCs showed timing differences consistent with a viscosity-mediated slowing of starch digestion [18]. A second randomized crossover study that quantified γ-PGA doses (approximately 58 mg vs. approximately 440 mg per 40 g) likewise reported lower early-phase glucose and insulin IAUCs with the high-γ-PGA natto [22]. Collectively, these acute data support postbiotic viscosity (γ-PGA) as a proximate mechanism for blunting postprandial excursions in humans.

Overall, the soy/legume ferment category illustrates how multiple levers—(i) isoflavone aglycone enrichment, (ii) peptide release (including DPP-IV-inhibitory sequences), (iii) microbiota remodeling, and (iv) viscosity-building postbiotics such as γ-PGA—can converge on glucose regulation. At the same time, effects on glycemic endpoints are more consistently documented for high-γ-PGA natto than for other soy/legume ferments, and human data outside acute meal tests remain limited. This heterogeneity motivates the more mechanistic treatment in Section 4 and Section 5 and the design recommendations in Section 7.

#### 3.1.3. Kombucha and Fermented Teas

Kombucha comprises a symbiotic consortium of yeasts and acetic acid bacteria (AAB) that remodel tea polyphenols and generate organic acids and cellulosic exopolymers [14]. Metagenomic and physicochemical profiling of commercial products consistently identifies *Komagataeibacter/Acetobacter* with yeasts in a cellulose pellicle, together with low residual sugars (e.g., glucose approximately 1.87 g/L, sucrose approximately 1.11 g/L, fructose approximately 0.05 g/L) and high total phenolics (approximately 290 mg gallic acid equivalents (GAE)/100 mL), indicating extensive carbohydrate utilization and polyphenol remodeling [67].

Untargeted metabolomics using ultrahigh-resolution Fourier Transform Ion Cyclotron Resonance Mass Spectrometry (FT-ICR-MS) has mapped yeast–yeast and yeast–AAB cross-feeding in simplified kombucha consortia (*Brettanomyces bruxellensis*, *Hanseniaspora valbyensis*, *Acetobacter indonesiensis*), assigning 506 features to unique molecular formulas (307 retained after replicate filtering) and identifying 136 “core metabolites” spanning Carbon, Hydrogen, and Oxygen/Carbon, Hydrogen, Oxygen, and Nitrogen/Carbon, Hydrogen, Oxygen, and Sulfur (CHO/CHON/CHOS) families; yeast–yeast coculture selectively increased peptide and fatty-acid signatures, whereas yeast–bacterium coculture shifted profiles toward organic-acid derivatives consistent with ethanol and sugar-acid oxidation [19]. A complementary FT-ICR-MS time course across production phases showed that the open-vessel acidification phase (P1) drove release/formation of gluconic acid (an AAB marker) and gallic acid, increased molecular diversity (e.g., 310 masses at day-7 black-tea kombucha vs. 138 in sugared black tea), and lowered pH from approximately 6.9 to approximately 4.2 with total acidity rising to approximately 20–41 meq/L; during the closed-vessel carbonation phase (P2), signals indicated consumption/transformation of oleic acid, and cellulose (1,4-β-glucan) signatures appeared as an additional AAB biomarker, with black-tea matrices showing more extensive polyphenol remodeling than green tea under comparable conditions [68]. Production-focused work with kombucha consortia also highlights BC as a salient postbiotic, with culture-system parameters modulating pellicle biosynthesis and properties [69].

Polysaccharide-focused work with mushroom-based kombucha variants (*Trametes versicolor*, *Lentinus edodes*) has revealed complex polysaccharide/phenolic signatures and in vitro immunomodulation in PBMCs (reduced Th2 cytokines and IL-10 without cytotoxicity up to 500 μg/mL), adding mechanistic context to kombucha’s postbiotic repertoire, although glycemic endpoints were not assessed [70].

In adults with T2D, a randomized, double-blind, crossover pilot (*n* = 12; 4-week periods with washout) reported that daily kombucha intake (approximately 240 mL/day) reduced fasting blood glucose from approximately 164 to 116 mg/dL by week 4 (*p* = 0.035), whereas a matched placebo decreased from approximately 162 to 141 mg/dL without statistical significance; reductions were larger among participants with baseline fasting glucose >130 mg/dL [71]. In healthy adults, an acute crossover meal study showed that consuming kombucha with a high-carbohydrate meal lowered both the glycemic index (GI) (to approximately 68) and the insulin index (to approximately 70) vs. control beverages (*p* = 0.041 for both), indicating attenuation of postprandial excursions [72]. These two studies together suggest that kombucha may exert detectable glycemic effects in the context of marked dysglycemia (T2D) or high-glycemic-load meals.

By contrast, an eight-week, parallel, RCT in free-living healthy adults deliberately maintained on a low-fiber, low-polyphenol “beige” Western diet assigned participants 2:1 to kombucha (*n* = 20 randomized; *n* = 16 analyzed) or control (*n* = 10; *n* = 8 analyzed). During the four-week intervention within this period, the kombucha group consumed one 16-oz bottle per day (two servings in total); clinic staff were blinded, while participants could not be. The primary endpoint was change in gut-microbiome composition; secondary endpoints included metabolic and inflammatory markers (e.g., IL-6, IL-10, C-reactive protein). Between-group differences in fasting glucose, fasting insulin, HOMA-IR, lipids, BP, and waist circumference were not significant. Within the kombucha group, fasting insulin and HOMA-IR increased over time (both *p* = 0.021), whereas the control group showed a decrease in high-density lipoprotein (HDL)-cholesterol (*p* = 0.042). Self-reported GI symptoms (diarrhea/bloating) were more frequent with kombucha (31.25% vs. 12.5%), and overall compliance was approximately 96%. Shotgun metagenomics nonetheless identified enrichment of *Weizmannia coagulans* and several SCFA-producing taxa in kombucha consumers. The trial was registered (NCT06484504) and industry-funded, and all participants remained on the beige-diet background throughout, underscoring how baseline diet and metabolic status may condition glycemic and microbiome responses to kombucha [73].

Longer-term data also differentiate kombucha based on tea type. In an 8-week pre–post intervention (*n* = 46; 23 normal-weight and 23 with obesity), daily black-tea kombucha (200 mL/day) was chemically profiled to contain 145 phenolic compounds (approximately 81% flavonoids, 19% phenolic acids). Microbiota shifts favored commensals, with increases in *Akkermansiaceae* and the butyrate-producer *Subdoligranulum* in the obesity group (*p* = 0.031), alongside reductions in obesity-associated genera (*Ruminococcus* and *Dorea*). Fungal diversity rose (↑*Saccharomyces*; ↓*Exophiala*, *Rhodotorula*), and *Pichia/Dekkera* emerged as biomarkers post-intervention. Although the design lacked a non-kombucha control and did not systematically assess glycemic endpoints, the directional changes support a microbiota signal under routine black-tea kombucha intake [74].

Over 10 weeks, an RCT during a calorie-restricted weight-loss program (control *n* = 29; kombucha *n* = 30) tested daily green-tea kombucha (200 mL/day). Between-group differences in intestinal permeability and fecal microbiota were not significant; however, the control group worsened on selected markers (e.g., lactulose:mannitol ratio 0.032 to 0.043, *p* = 0.01; fecal pH 7.30 to 7.07, *p* = 0.04), whereas the kombucha group reported better gastrointestinal symptoms and showed distinct post-intervention serum-metabolome features. Within-group SCFAs declined in both arms (e.g., fecal butyrate: control 10.193 to 8.674 µg/g, *p* = 0.02; kombucha 9.129 to 8.005 µg/g, *p* = 0.01). Taken alongside the black-tea pre–post study, these findings suggest that black- and green-tea kombuchas may exert partly distinct microbiota and metabolome signatures in humans, but robust, tea-type–specific effects on glycemic markers have yet to be demonstrated [75].

In diet-induced obese mice with Non-alcoholic fatty liver disease (NAFLD) features, kombucha supplementation improved oral glucose tolerance and reduced fasting hyperinsulinemia; in the liver it lowered citrate synthase and phosphofructokinase-1 activities, downregulated bile acid–sensing receptors TGR5 and FXR at the gene-expression level, attenuated steatosis and collagen deposition, and restored acute insulin-induced protein kinase B (Akt) serine phosphorylation—indicating coordinated effects on hepatic carbohydrate and bile acid metabolism with histologic improvement [76]. A complementary high-fat, high-fructose rat model showed that 10 weeks of green- or black-tea kombucha with the obesogenic diet improved glucose metabolism, reduced circulating triglycerides and adiposity, and reversed liver steatosis while mitigating oxidative stress and inflammatory read-outs [77]. These mechanistic insights are currently confined to preclinical models and inform hypotheses about bile-acid and redox signaling (FXR/TGR5, AMPK/Nrf2) that require further testing in humans (Section 5).

Overall, kombucha emerges as a tea-based ferment with a *Komagataeibacter*–yeast core and an organic-acid/phenolic/cellulosic postbiotic repertoire. Human data suggest glycemic benefits in adults with T2D and during high-glycemic-load meals, but neutral or even adverse metabolic signals in some healthy cohorts. Comparative data on black-vs. green-tea kombucha in humans are still limited and indirect: Black-tea kombucha has mainly been linked to microbiota shifts in pre–post designs, whereas green-tea kombucha has been studied within hypocaloric diets with modest or null effects on gut permeability and composition. Across studies, a persistent limitation is the lack of standardized dosing (volume, fermentation time), organic-acid and ethanol profiling, and quantification of key polyphenols and postbiotic polymers, which hampers comparisons and attribution of effects. Taken together, current evidence indicates that kombucha’s metabolic impact is context dependent and shaped by dose (approximately 240–330 mL/day), fermentation chemistry (including BC and organic-acid output), tea type, and background diet/metabolic status [78].

#### 3.1.4. Plant-Based Kefir and Analogues

Water kefir is a sucrose-based fermentation in which translucent kefir grains act as living exopolysaccharide biofilms that host LAB, AAB, and yeasts; grain composition and metabolite output vary with substrate, mineral content, and temperature across producers and batches [15]. Detailed structural analysis of water kefir EPS shows that the insoluble grain polymers are predominantly dextran with an α-(1→6) backbone and O-3 branching, containing a higher proportion of 1,3-linked glucose units than the soluble fraction, whereas the beverage contains structurally distinct EPS—features consistent with contributions from multiple bacterial producers [20].

In practice, dextran synthesis by dextransucrase-positive *Liquorilactobacillus hilgardii* and related taxa scaffolds grain formation and growth. Time-resolved work tracking a 192 h fermentation showed that sucrose was completely consumed by 24 h, coinciding with the main burst of grain polysaccharide production; ethanol and lactic acid were the dominant metabolites, whereas glycerol, acetic acid, and mannitol accrued at lower levels, with most metabolite formation occurring within the first 72 h as pH fell from approximately 4.26 to approximately 3.45. The grain-associated microbiota comprised *Lactobacillus casei/paracasei*, *L. harbinensis*, *L. hilgardii*, *Bifidobacterium psychraerophilum/crudilactis*, *Saccharomyces cerevisiae*, and *B. bruxellensis*, alongside a volatile profile dominated by ethyl acetate, isoamyl acetate, ethyl hexanoate, ethyl octanoate, and ethyl decanoate [79]. Complementarily, shotgun metagenomics at 24 and 72 h (7.1% sucrose; 17.6% fig extract; 21 °C) identified *L. hilgardii*, *L. harbinensis*, *L. nagelii*, *L. paracasei*, a *Lactobacillus hordei/mali*–like species, *Bifidobacterium aquikefiri*, and the yeasts *S. cerevisiae* and *B. bruxellensis*, and uncovered a novel *Oenococcus* (*Candidatus Oenococcus aquikefiri*). Functional binning linked mannitol-from-fructose production to *L. hilgardii*, *Candidatus O. aquikefiri*, and *B. aquikefiri*, assigned dextransucrase genes to *L. hilgardii*, *L. hordei/mali*, and *Candidatus O. aquikefiri*, and highlighted cross-feeding (e.g., amino acid/cofactor supply) within the consortium [80].

Typical water-kefir fermentations yield lactic and acetic acids, carbon dioxide, trace ethanol, glycerol, and mannitol; the community structure and metabolite output vary with grain provenance, substrate (sugars and fruit additives), mineral content, and temperature across producers and batches [15,81,82]. In flavored systems, physicochemical and sensory trajectories—including pH and titratable acidity, residual sugars/ethanol, organic acids, volatile esters, color, and consumer liking—diverge by matrix and processing, underscoring matrix-driven variability relevant to translation work [83].

In soymilk kefir, fermentation increases β-glucosidase activity and shifts the isoflavone pool toward aglycones. Across ten soybean cultivars, aglycone daidzein increased from 17.35 to 60.15 μg/g in unfermented soymilk to 23.79–91.03 μg/g after 24 h of kefir fermentation; glycitein was not detected post-fermentation, while malonyl and acetyl conjugates declined. In the Guixia 2 cultivar, the sum of individual aglycones reached 72.07 ± 0.53 μg/g with selected starters, whereas total isoflavones showed a small reduction overall [84]. In parallel models, fermenting soymilk with *L. plantarum* LP95 produced a sustained acidic, antioxidant-enriched matrix (pH 6.5 to 4.2 in 24 h; viable counts >7 log CFU/mL through 49 days at 4 °C) with higher Trolox-equivalent antioxidant capacity and lower Thiobarbituric acid-reactive substances (TBARS) during storage, illustrating a stable postbiotic milieu in plant-based substrates [85].

Mechanistically, water-kefir LAB (for example, *L. hilgardii*) synthesize dextran and other branched glucans that increase viscosity and may slow starch digestion [15,20]. In plant-milk kefir, fermentation with kefir cultures produces α-galactosidase and β-glucosidase that hydrolyze raffinose and stachyose (reported up to approximately 100% and approximately 92%, respectively) and convert isoflavone glycosides to aglycones [86]; recent overviews of plant-based kefir systems also document EPS outputs, enzyme activities, and amylase-inhibitory signals across plant matrices, reinforcing the mechanistic plausibility for early postprandial attenuation [87]. These matrix- and strain-level differences mirror the physicochemical/sensory variability captured in flavored water-kefir models [83] and motivate standardized characterization in future human trials.

Human trials directly testing non-dairy/plant-based kefir for glycemic endpoints remain limited. Evidence from broader kefir meta-analyses—dominated by dairy kefir—suggests reductions in fasting blood glucose (weighted mean difference (WMD) −10.28 mg/dL, 95% CI −16.53 to −4.02; *p* = 0.001) and insulin (WMD −2.87 μU/mL, 95% CI −3.96 to −1.78; *p* < 0.00001), with no significant change in HbA1c overall [88]. We include one dairy kefir RCT in T2D (8 weeks, 600 mL/day) as a mechanistic benchmark—reporting greater reductions in fasting glucose (*p* = 0.01 between groups) and HbA1c (*p* = 0.02 between groups)—while noting that milk-specific components and matrix properties limit direct extrapolation to plant-based kefirs [89].

In high-fat/high-fructose diet (HFFD) rats, kefir-fermented soy milk inhibited digestive enzymes in vitro (α-amylase half-maximal inhibitory concentration (IC50) approximately 52.7 μg/mL; pancreatic lipase IC50 approximately 39.4 μg/mL) and, in vivo, lowered fasting blood glucose (approximately −36% vs. HFFD control), reduced intestinal and pancreatic lipase activities by approximately 26–35%, improved the lipid profile (↓Total Cholesterol (TC), ↓LDL; ↑HDL), and mitigated hepatic and renal injury markers—supporting a functional, enzyme-targeted route for glycemic improvement in a preclinical model [32,90]. In streptozotocin–nicotinamide diabetic rats, a goat- and soy-milk kefir combination produced a greater fall in plasma glucose than goat-milk kefir alone, increased glutathione peroxidase activity, and improved islet morphology with β-cell counts approaching those of non-diabetic controls [91].

Overall, plant-based kefir provides a reproducible LAB/AAB/yeast consortium embedded in dextran-rich grains, generates organic acids and EPS with plausible glycemic relevance, and increases isoflavone aglycones in soy matrices. At the same time, human glycemic evidence specific to non-dairy kefir is sparse and largely indirect, relying on extrapolation from dairy kefir and preclinical plant-based models. This defines a tractable gap that we revisit in Section 7 and in the recommendations for future trials.

#### 3.1.5. Other Plant Matrices (Fermented Cereals and Pulse-Based Sourdoughs)

Sourdough fermentation broadens the plant-based portfolio by pairing LAB and yeasts to acidify cereal and pulse doughs, degrade phytate, remodel starch–protein–fiber networks, and accumulate organic acids—changes that collectively tend to slow starch hydrolysis and attenuate postprandial glycemic excursions. Comparative product testing generally indicates that sourdough breads display lower estimated GI than comparable yeast-leavened breads of similar flour composition, consistent with matrix-driven glycemic attenuation [16].

Formulation can further potentiate these effects. In a randomized crossover clinical trial, jabuticaba-peel-enriched sourdough bread increased fiber from approximately 1.0 to 2.3–2.9 g/100 g, raised antioxidant capacity 1.35–3.53× and total reducing capacity 1.56–2.67× vs. control, and maintained a 7-day shelf-life. Postprandially, the control bread elicited a glycemic peak at 30 min that persisted to 45 min, whereas the jabuticaba-peel sourdough produced a less prominent peak at 45 min with a smoother decline to 180 min; serum antioxidant capacity also rose by 3 h, while satiety profiles were broadly similar, with slightly higher satiety and lower desire to eat at 60 min for the enriched sourdough [92]. This study illustrates how combining sourdough fermentation with polyphenol- and fiber-rich by-products can shape both glycemic and redox trajectories, but its findings are not directly generalizable to simpler white-flour sourdoughs.

At the ingredient level, red-bean sourdoughs fermented with single (*Lactobacillus fermentum*) or mixed starters *(L. fermentum* + *Kluyveromyces marxianus*)—with or without wheat bran—showed significant reductions in tannins, phytic acid, and trypsin inhibitors, and increases in total phenolics, flavonoids, gallic acid, and soluble fiber; mixed-starter systems delivered the largest shifts, consistent with enhanced β-glucosidase, feruloyl esterase, and phytase activities. Bread quality also improved (higher specific volume, lower crumb firmness, better sensory scores). In mice, diets incorporating mixed-starter red-bean-plus-bran sourdough decreased serum pro-inflammatory cytokines, improved the HDL/LDL ratio, and enhanced glucose tolerance and insulin sensitivity; gut-microbiota diversity increased, with higher *Bifidobacterium* abundance and greater SCFA production vs. controls and white-bread comparators, supporting a microbiota-linked route to metabolic benefit [93].

Preclinically, feeding two sourdough breads to streptozotocin–nicotinamide T2D rats for one month improved hyperglycemia and oral glucose tolerance, favorably shifted oxidative and inflammatory markers, lipids, and liver/kidney function, and enhanced cognitive performance. The sourdough bread richer in unsaturated fatty acids (approximately 79.33% of total fatty acids) outperformed the higher-saturated-fatty-acid loaf (approximately 16.08% saturated fatty acids). Mechanistically, hippocampal Brain-Derived Neurotrophic Factor (BDNF) and Nrf2 expressions increased, while hepatic glucose transporter 2 (GLUT2) and phosphoenolpyruvate carboxykinase (PEPCK) were down-regulated, consistent with improved glucose handling and neuroprotection in this animal model [94].

Across cereals and pulses, sourdough processes offer a controllable LAB–yeast ecology that, via acidification, phytate degradation, network remodeling, and strategic co-delivery of fiber and polyphenols, can reduce predicted or acute glycemic impact in humans and improve glycemic endpoints in rodents. However, recent structured and systematic reviews of sourdough breads and low-GI products produced with microbial fermentation underline that these benefits are highly heterogeneous and strongly conditioned by flour type (wholegrain vs. refined), particle size, fermentation protocol (traditional, long-fermented Type I vs. shorter, industrial Type II systems), dough hydration, and co-ingredients [25,34]. Randomized trials in adults at risk for T2D, for example, show that modifying wholegrain flour particle size within bread formulations can yield only small or neutral changes in postprandial glucose and insulin responses, highlighting that flour characteristics alone do not guarantee clinically meaningful glycemic improvements [95]. Mechanistic work comparing spontaneous sourdough and solid-state fermentation of whole wheat further demonstrates that different fermentation strategies markedly alter acidification dynamics, protein network structure, and phenolic/tannin profiles—matrix properties that are directly relevant to starch digestibility and thus to glycemic response [96]. A broader overview of microbe-enabled low-GI cereal and legume products similarly concludes that the magnitude of GI reduction depends not on “sourdough” labeling per se, but on a combination of flour selection, fermentation time–temperature, and formulation [97]. To enable comparability across styles and co-ingredients, future human trials should therefore report, at minimum, flour type and cultivar, fermentation time and temperature, dough pH and titratable acidity, organic-acid profiles, in vitro starch digestibility metrics, and standardized postprandial endpoints, and a clear specification of whether Type I or Type II sourdough systems are used.

## 4. Microbial Biotransformation of Phytochemicals

Fermentation—understood as desired microbial growth and enzymatic conversion of food components—provides a biochemical setting in which microbes reshape plant matrices beyond simple preservation. In this context, microbial enzymes such as β-glucosidases, tannases, esterases, and phenolic-acid decarboxylases convert bulky polyphenol glycosides and other conjugates into smaller, more absorbable molecules, while generating new derivatives that are scarce or absent in the raw substrate. These transformations recur across vegetable-, soy-, tea-, and cereal/pulse-based ferments and are central to how fermentation can influence host metabolism [6,7,8]. In parallel, consortia-level cross-feeding produces organic acids and other low-molecular-weight metabolites that reshape the chemical milieu (pH, redox, osmolarity) and can interface with host pathways; analogous transformations and metabolite classes have been mapped in fermented foods with direct relevance to microbe–microbe and microbe–host interactions [11]. In the ISAPP sense, many of these inanimate microbial cells, cell fragments, and metabolites constitute a postbiotic “payload” within the fermented matrix, even when live microorganisms are still present.

A consistent pattern emerges most clearly in soy- and tea-based systems: Glycosylated isoflavones and flavonoids are hydrolyzed to their aglycones, which display higher bioaccessibility and distinct biological activity in vitro and in vivo; at the same time, microbial cross-feeding yields organic acids and low-molecular-weight phenolics that further remodel the chemical milieu. In fermented soymilk, aglycone enrichment tracks with measurable β-glucosidase activity, and kombucha metabolomics map complementary yeast–bacterium interactions that produce acetic, gluconic, and lactic acids alongside remodeled polyphenols [19,84,85], consistent with metagenomic/physicochemical profiles showing low residual sugars and high phenolic content [67]. Comparable biotransformation patterns are now being described in cereal and pulse matrices, where lactic fermentations generate overlapping clusters of organic acids, phenolic derivatives, peptides, and exopolymers at the point of consumption. Together, these changes plausibly underpin reported improvements in redox balance and inflammatory tone, and provide the biochemical backdrop for the polyphenol-focused routes in Section 4.1, the postbiotic-metabolite and polymer pathways in Section 4.2, and the downstream insulin-sensitivity developed in Section 5 and Section 6.

### 4.1. Deglycosylation of Polyphenols: From Glycosides to Aglycones

#### 4.1.1. Enzymatic Framework and General Pattern

Fermentation supplies a relatively well-defined enzymatic toolkit that remodels plant polyphenols. Microbial β-glucosidases hydrolyze O-glycosides (e.g., rutin → quercetin; naringin → naringenin), tannases cleave galloyl and depsidic bonds (liberating gallic acid), esterases de-esterify phenolic esters (e.g., chlorogenic → caffeic + quinic acids), and phenolic-acid decarboxylases convert hydroxycinnamates into vinyl derivatives (e.g., p-coumaric → 4-vinylphenol; ferulic → 4-vinylguaiacol). Contemporary syntheses consistently describe this pattern across vegetable-, soy-, and tea-based ferments, together with concomitant rises in total phenolics and in vitro radical-scavenging capacity [6].

As a primary quantitative example comes from the fermentation of sea-buckthorn leaves with a β-glucosidase-producing *Eurotium amstelodami* strain (BSX001): Rutin decreased from 4.61 ± 0.13 to 0.92 ± 0.05 mg/g dry weight, while total phenolics increased from 55.97 ± 1.72 to 100.16 ± 3.25 mg GAE/g dry weight; aglycones (quercetin, kaempferol, isorhamnetin) rose in parallel and 2,2-diphenyl-1-picrylhydrazyl/2,2′-azino-bis (3-ethylbenzothiazoline-6-sulfonic acid)/ferric reducing antioxidant power (DPPH/ABTS/FRAP) readouts improved, documenting enzyme-linked deglycosylation and enhanced redox activity rather than simple concentration effects [98].

Although most of these data derive from in vitro or matrix-focused studies, the pattern is mechanistically relevant for glycemic control: Aglycones and low-molecular-weight phenolics differ from their parent glycosides in absorption, tissue distribution, and signaling properties, and can interface with redox-inflammation pathways and incretin biology. In later sections we explore how these fermentation-driven changes in phenolic profiles may contribute—alongside organic acids and polymers—to improved insulin sensitivity and postprandial glycemic handling, while noting that direct human evidence linking specific enzymatic transformations to glycemic endpoints remains limited.

#### 4.1.2. Soy/Plant-Milk Systems (Isoflavone Deglycosylation)

In soy matrices, kefir-style and related fermentation consistently shift the isoflavone pool toward aglycones via microbial β-glucosidases. In a ten-cultivar screen, soymilk fermented for 24 h increased aglycone daidzein from approximately 17.35–60.15 μg/g (unfermented) to approximately 23.79–91.03 μg/g, while malonyl/acetyl conjugates declined and glycitein frequently fell below detection—patterns that reflect targeted enzymatic remodeling rather than simple concentration effects, with magnitudes dependent on cultivar and starter choice [84]. This aglycone enrichment is mechanistically relevant because aglycones exhibit higher intestinal bioaccessibility and distinct signaling properties compared with their parent glycosides, offering plausible links to incretin biology, redox–inflammatory tone, and ultimately insulin sensitivity, even though direct glycemic readouts are seldom reported in these analytical studies.

In a complementary plant-milk model, fermenting soymilk with *L. plantarum* LP95 yielded a refrigerated beverage that maintained viable counts >7 log CFU/mL over extended storage, displayed higher Trolox-equivalent antioxidant capacity, and exhibited lower lipid peroxidation vs. unfermented control [85]. Together with the isoflavone data, these findings support a stable, fermentation-derived postbiotic milieu in soy-based drinks that can sustain aglycone exposure and enhanced antioxidant capacity throughout shelf-life. At present, most of this evidence is matrix- and mechanism-focused; in subsequent sections we explore how similar deglycosylation patterns, when integrated with viscosity-building polymers and organic acids in whole meals, may contribute to the modest, context-dependent glycemic effects observed in human trials of fermented soy foods.

#### 4.1.3. Tea Ferments (Tannase-Mediated Release of Phenolic Acids; Organic-Acid Formation)

In kombucha and related tea ferments, microbial tannase and esterases liberate gallic acid from gallated catechins, while yeast–AAB cross-feeding oxidizes sugars and ethanol to organic acids such as acetic, gluconic, and lactic acids. Untargeted FT-ICR-MS in simplified consortia (e.g., *B. bruxellensis, H. valbyensis, A. indonesiensis*) has mapped these trajectories and the expansion of molecular diversity during acidification, with signatures of gluconic and gallic acids consistent with tannase activity and AAB-driven sugar-acid oxidation (as detailed in Section 3.1.3) [19]. Metagenomic and physicochemical profiling of commercial kombuchas corroborates a *Komagataeibacter/Acetobacter*–yeast core with low residual sugars and high total phenolics, indicating extensive substrate turnover and polyphenol remodeling [67]. Under comparable conditions, black-tea matrices tend to show more extensive polyphenol transformation than green tea, suggesting that tea type modulates the balance between catechin degradation, phenolic-acid release, and organic-acid accumulation.

Process-oriented studies reinforce and extend this biochemical framework. In a metagenomic–organoleptic evaluation substituting conventional black-tea extract with recycled plant substrates, Selvaraj and colleagues characterized both the Symbiotic Culture of Bacteria and Yeast (SCOBY) and the beverage: *Komagataeibacter*/*Acetobacter* cores with yeast partners were consistently present; fermentation reduced pH and increased titratable acidity, while total phenolics and antioxidant readouts (e.g., DPPH/ABTS) rose in parallel with substrate turnover [99]. Importantly, hedonic scores were maintained across formulations despite shifts in substrate and metabolite profiles, indicating that substantial biochemical remodeling—tannase-mediated release of phenolic acids and altered organic-acid profiles—can occur without compromising sensory acceptance.

Matrix and storage conditions further shape the organic-acid and phenolic landscape. In a soursop kombucha model, response-surface optimization highlighted 25 °C and 14 days as a suitable fermentation window; subsequent storage experiments showed that antioxidant activity peaked at 14 days at 25 °C under light, whereas total phenolics were highest at 7 days at 4 °C in the dark. Sugars declined over storage, ethanol dropped markedly, and organic acids (e.g., acetic, gluconic, malic) varied by condition [100]. When kombucha was spray-dried and encapsulated in gum arabic, retention of organic acids and antioxidant and antibacterial activities during storage was superior to that of the non-encapsulated beverage [101]. These findings illustrate how post-fermentation handling can substantially modify the apparent postbiotic profile, even when the starting fermentation is similar.

Pragmatically, these data argue that authors should report, at minimum, the substrate identity and tea type (black, green, mixed; with approximate polyphenol load, e.g., mg GAE/L), SCOBY/inoculum mass, fermentation time and temperature, and storage or encapsulation conditions, because these variables demonstrably shift sugar–acid trajectories, phenolic retention, antioxidant/antibacterial activity, and sensory readouts [100,101]. While most of these studies do not include direct glycemic endpoints, they define a mechanistic—tannase-mediated phenolic-acid release, catechin degradation, and organic-acid/cellulosic postbiotic production—that constrains how kombucha and other tea ferments might influence glucose metabolism in the human trials discussed in Section 3.1.3 and Section 6.

#### 4.1.4. Vegetable Ferments (Kimchi/Sauerkraut: Phenolic Remodeling, Sugar Alcohols, GABA)

Vegetable lactic fermentations remodel *Brassica*/cabbage matrices via LAB-dominated successions and generate characteristic small molecules beyond simple preservation. In artisanal sauerkraut from two small-scale producers, pH dropped rapidly within the first week (from 5.7 ± 0.2 to 3.8 ± 0.1 in SK1 and 5.7 ± 0.3 to 4.0 ± 0.02 in SK2), while LAB counts increased from approximately 3.4–3.6 to approximately 5.7–8.1 log CFU/mL by day 7 and reached 6.4 ± 0.5 (SK1) and 8.2 ± 0.2 (SK2) log CFU/mL by day 35. Community succession proceeded from early *L. mesenteroides/pseudomesenteroides* to later *L. plantarum/paraplantarum*, with 220 LAB isolates recovered overall; ^1^H-NMR fingerprints highlighted lactate and acetate among dominant low-molecular-weight features, and sauerkraut brines modulated cytokine responses (↑TNF-α/IL-6 with compensatory ↑IL-10 at 24 h) in a PBMC–Caco-2 co-culture—consistent with postbiotic-driven immunomodulation alongside organic-acid formation rather than a purely preservative effect [61].

In kimchi, sugar-alcohol and GABA pathways illustrate strain-level cross-feeding during lactic fermentation. A serial co-fermentation using *L. citreum* S5 followed by *L. plantarum* KS2020 produced a GABA-enhanced kimchi beverage, with viable counts of approximately 9.11–9.42 log CFU/mL on day 1 at 5% sucrose; mannitol synthesized by *Leuconostoc* was subsequently depleted as *L. plantarum* grew, documenting dynamic fructose-reduction → mannitol fluxes together with amino-acid decarboxylation in a plant matrix [51]. In parallel, starter selection of commercial kimchi manufacture using *L. mesenteroides* strains underscores mannitol-producing and EPS-forming phenotypes that co-define texture and metabolite outputs relevant to downstream function [53]. Mechanistically, kimchi-derived strains can also attenuate starch digestion in vitro: *L. plantarum* LRCC5314 inhibited α-amylase by approximately 72.9% and α-glucosidase by approximately 51.2%, providing a plausible interface between the fermentation-derived chemical milieu and early postprandial glycemic handling [55].

GABA production in kimchi is therefore best viewed as part of a broader postbiotic signature that includes organic acids, sugar alcohols, and EPS. While GABA has been linked to neuroendocrine and blood-pressure effects in preclinical models, its direct contribution to glycemic control in humans consuming kimchi remains poorly characterized and is often inferred indirectly from animal data or from studies using isolated GABA rather than food-based sources. In contrast, organic acids, mannitol, and EPS from vegetable ferments have clearer mechanistic links to gastric emptying, enzyme inhibition, osmotic effects, and barrier/inflammatory signaling, providing more immediate routes to explain the modest but measurable glycemic and metabolic signals observed in kimchi trials (Section 3.1.1).

Taken together, vegetable ferments such as sauerkraut and kimchi generate organic acids (lactate, acetate), sugar alcohols (mannitol), GABA, and strain-dependent EPS, superimposed on phenolic remodeling, with microbial succession, recipe, and starter choice determining the balance of these outputs. These low-molecular-weight acids, osmolytes, and biogenic amines form part of a postbiotic repertoire that can influence viscosity, barrier integrity, and host inflammatory tone—setting the biochemical context for the mechanistic pathways developed in Section 4.2 and their translation to glycemic outcomes in Section 5 and Section 6.

#### 4.1.5. Cross-Matrix Synthesis and Analytical Considerations

Across vegetables, soy, tea, and cereal/pulse doughs, a recurring analytical theme is that substrate, inoculum, and processing choices reshape both microbial consortia and the small-molecule landscape, which in turn conditions host-facing outcomes. Recent work on lactic acid bacteria-fermented cereal-based foods explicitly frames these low-molecular-weight acids, EPS, peptides, and cell-wall-derived fragments as a postbiotic repertoire, and links their antioxidant, anti-inflammatory, and enzyme-inhibitory activities to specific fermentation parameters and analytical markers, reinforcing the need to co-characterize chemistry and function in plant-based ferments [102]. Kombucha illustrates this dependency particularly well: Metagenomic and untargeted metabolomic work shows yeast–AAB cross-feeding with extensive substrate turnover, yet residual sugars, organic acids, and phenolics vary by tea type, inoculum mass, and fermentation/finishing steps. These data underscore the need to co-report substrate/tea specification, SCOBY mass, time–temperature, pH/titratable acidity, residual sugars (g/L), organic-acid profile (e.g., HPLC), ethanol %, and cellulose pellicle metrics alongside microbiome readouts when linking chemistry to physiology [19,67]. Production-focused studies likewise demonstrate that BC is a substantial, process-sensitive postbiotic in kombucha, arguing for its quantification when interpreting mechanistic and clinical findings [69].

Convergence across matrices is also evident at the consortia–metabolite–host interface. In preclinical work, kombucha-associated microbes reprogram host metabolic pathways and suppress lipid accumulation, aligning with the organic-acid/phenolic signatures observed in compositional studies and motivating paired host omics (transcriptome and metabolome) in future human trials [103]. Clinically, black- and green-tea kombucha interventions have reported shifts in gut microbiota composition, serum metabolome, and GI symptoms in adults with and without obesity [74,75]. However, systematic appraisals on kombucha trials consistently emphasize heterogeneity in dose, duration, product chemistry, and endpoints, making it difficult to integrate findings across studies [78,104]. Accordingly, cross-matrix studies should pair microbiome and host-omics endpoints with a minimal analytical panel—polyphenols (e.g., mg GAE/L), organic acids, residual sugars and ethanol, cellulose/pellicle metrics where applicable, viable counts, serving dose and frequency, and background diet—so that microbiota and metabolic outcomes can be meaningfully compared across fermented foods.

Downstream handling further modulates metabolite stability and bioactivity. Encapsulation or spray-drying with gum arabic can better preserve organic acids and antioxidant capacity during storage than the unencapsulated beverage, whereas storage time, temperature, and light materially shift sugar–acid profiles and functional readouts in fruit-tea kombuchas [83,99]. These observations generalize to other plant matrices and argue for explicit reporting of finishing, packaging, and storage conditions in the fermented products used for test meals and interventions, rather than assuming that freshly fermented and shelf-stored products are equivalent.

A second cross-matrix theme is postbiotic flux into the host. In a quantitative screen of foods and beverages, acetate emerged as the dominant SCFA; per serving, kombucha provided one of the highest oral acetate (approximately 1226 mg acetate per 330 mL bottle), alongside vinegars, which delivered several hundred to approximately 700–1018 mg acetate per tablespoon (e.g., apple cider vinegar approximately 1016 mg), placing them among the richest oral SCFA sources. In a pharmacokinetic study in healthy adults, a drink containing approximately 25.8 mmol acetate increased plasma acetate from approximately 121.3 to approximately 263.5 μmol/L at 60 min and raised acetate AUC (12,346 vs. 1292 μmol/L·min vs. control), while propionate AUC also rose despite minimal propionate in the drink—suggesting endogenous contributions [105]. Methodological notes from this work highlight lot-to-lot variability, potential analytical artifacts (e.g., tube contamination), and the need for 13C-labeled tracers and diet records, because matrix and co-ingested foods modulate absorption and systematic appearance. For fermented plant foods, this implies that postprandial glucose/insulin iAUCs should be paired with circulating SCFAs (ideally with isotopologues) and detailed reporting of beverage/food chemistry and background diet to distinguish exogenous SCFA delivery from microbiota-mediated endogenous production.

Quality and safety analytics intersect with mechanistic interpretation. Head-to-head testing of three kombucha cultures showed that concentrated fermented teas and derived vinegar exerted bactericidal activity comparable to reference antibiotics (approximately 1 mmol ampicillin against *Escherichia coli* or 0.01 mmol penicillin against *Staphylococcus epidermidis*); neutralizing the pH abolished these effects, indicating acid-driven killing [106]. Inoculation with macerated pellicles accelerated growth and acidification, and sucrose/glucose/fructose supported robust growth, whereas maltose/galactose/lactose performed poorly and were prone to mold contamination [106]. Separately, SCOBY-derived isolates degraded aflatoxin B1 in vitro in spiked media, with degradation dependent on the isolate and assay conditions, underscoring a potential biotransformation capacity within kombucha microbiota. Translational work should therefore specify starting contaminant loads, pH/time windows, and identify degradation products before inferring safety benefits [107]. More broadly, probiotic and bioprocess characterization frameworks recommend standardized strain-level annotation, viability/CFU reporting, and multi-omics-based function attribution [108], all of which are directly applicable to fermented-food trials that aim to link microbial biotransformation to glycemic endpoints.

At a minimum, plant-ferment studies should therefore report the following information: substrate identity/cultivar; inoculum source and mass (or strain-defined starters); fermentation time–temperature; pH and titratable acidity; residual sugars and organic acids (e.g., by HPLC); ethanol %; EPS/cellulose (g/L or pellicle mass/thickness where relevant); viable LAB/AAB/yeast counts; key phytochemical markers (e.g., isoflavone aglycones in soy; phenolic acids in tea; phytate reduction in cereals); finishing/packaging and storage conditions; background diet controls; and paired endpoints (glycemic iAUCs, incretins, circulating SCFAs, and microbiome/host-omics). This common grid—outlined conceptually in Table 1 and Table 2—will enable cleaner cross-matrix comparisons and more robust mechanistic attribution across vegetables, soy, tea, and cereal/pulse ferments. These proposed items are consistent with recent postbiotic-focused frameworks for LAB-fermented cereal foods, which likewise call for joint reporting of postbiotic profiles and bioactivity readouts in fermented plant matrices [102].

### 4.2. Enzyme-to-Metabolite Routes with Glycemic Relevance

#### 4.2.1. Canonical Enzymatic Routes: From Glycosides/Esters to Aglycones and Vinyl Phenols

Across plant fermentations, polyphenol remodeling follows reproducible, enzyme-mediated routes. β-Glucosidases from LAB and yeasts cleave O-glycosidic bonds to liberate flavonoid aglycones (for example, rutin → quercetin; naringin → naringenin), increasing lipophilicity and intestinal bioaccessibility [109,110,111]. Operationally, food-grade β-glucosidases show pH optima around 4–6 and temperature optima near 30–40 °C, with certain wine-associated strains retaining activity under ethanol—conditions compatible with real food fermentations [110]. In soy models, fermentations with *Lactobacillus/Bifidobacterium* increase isoflavone aglycone fractions within 12–48 h, consistent with measurable β-glucosidase activity rather than simple concentration effects [111]. In leaf-based systems, controlled fungal–microbial fermentation yields quantitative shifts: Rutin decreases (approximately 4.61 → 0.92 mg/g dry weight) as quercetin/kaempferol rise, total phenolics nearly double (approximately 56 → 100 mg GAE/g DW) and DPPH/ABTS/FRAP readouts improve—direct evidence of enzyme-linked remodeling in a food-relevant matrix [112].

Food-grade esterases from LAB enact bona fide de-esterification of phenolic esters. A purified chlorogenate-active esterase from *Lactobacillus helveticus* efficiently hydrolyzes 5-caffeoylquinic acid (5-CQA, chlorogenic acid) to caffeic and quinic acids under mildly acidic, fermentation-relevant conditions and remains active in a real food matrix (defatted sunflower meal), where application lowered chlorogenic acids and prevented chlorogenic-acid-quinone (CGAQ)-driven greening—demonstrating in situ de-esterification rather than artefactual degradation [113]. Complementary surveys catalog microbial tannases and chlorogenate/phenolic esterases that cleave galloyl/depsidic and caffeoyl–quinic linkages and outline pH/temperature optima typical of fermented foods; structural work on the *L. helveticus* esterase delineates the catalytic pocket accommodating 5-CQA, providing a mechanistic basis for chlorogenic-acid → caffeic + quinic conversion in food systems [114]. Together, these lines of evidence establish the ester-to-acid trajectory as a canonical route that generates smaller, more bioaccessible phenolic acids with distinct biological profiles.

Phenolic-acid decarboxylases (PADs) then convert hydroxycinnamates into vinyl derivatives (for example, p-coumaric → 4-vinylphenol; ferulic → 4-vinylguaiacol), yielding smaller, more hydrophobic products with distinct sensory and biological footprints; cell-free PAD systems from food-grade microbes catalyze these reactions under mildly acidic conditions typical of vegetable and cereal ferments [115]. Collectively, the β-glucosidase/tannase–esterase/PAD triad provides a mechanistic scaffold for the glycoside-to-aglycone, ester-to-acid, and acid-to-vinyl-phenol trajectories that underpin the smaller-molecule, higher-bioaccessibility profiles repeatedly observed in fermented plant foods [84,113,115,116].

From a glycemic standpoint, these routes are best viewed as enabling mechanisms: They increase the pool of readily absorbable phenolics that can modulate redox status, endothelial and inflammatory signaling, and incretin pathways (see Section 5.2 and Section 5.3), but direct links to improved postprandial glycemia in humans remain largely inferential and are often supported by in vitro and animal models. In the following subsections, we connect these enzymatic patterns to specific matrices (soy/plant milks, tea, vegetables) and to downstream metabolites—including SCFAs and bile-acid-related products—that have more immediate proximity to glucose control.

#### 4.2.2. Soy/Plant-Milk Systems: Isoflavone Deglycosylation and β-Glucosidase Activity

In soy matrices, fermentation consistently increases β-glucosidase activity and shifts the isoflavone pool toward aglycones. In a kefir-style soymilk model spanning ten soybean cultivars, aglycone enrichment was evident within 24 h: Daidzein rose from 17.35–60.15 μg/g (unfermented) to 23.79–91.03 μg/g after fermentation, while malonyl/acetyl conjugates declined and glycitein frequently became undetectable—demonstrating enzyme-linked remodeling rather than simple concentration effects [84]. In parallel, a plant-milk fermentation with *L. plantarum* LP95 produced a durable postbiotic matrix through cold storage (pH 6.5 → 4.2 in 24 h; viable counts > 7 log CFU/mL maintained for 49 days at 4 °C) with higher Trolox-equivalent antioxidant capacity and lower TBARS, supporting sustained aglycone exposure within a stable, acidified environment [85].

Beyond kefir grains, food-grade starter choices and analytics also govern the extent of deglycosylation. Standardized quantification in fermented plant milks reports net increases in genistein and daidzein with concomitant losses of malonyl forms, and highlights starter composition and cultivar as primary drivers of variability in 24–48 h processes [117].

A complementary ester route operates alongside β-glucosidases. A purified chlorogenate-active esterase from *L. helveticus* efficiently hydrolyzes 5-CQA into caffeic and quinic acids under mildly acidic, fermentation-relevant conditions and remains active in a real food matrix (defatted sunflower meal), where its application decreased chlorogenic acids and prevented CGAQ-driven greening—direct evidence of in situ de-esterification [118]. Surveys of food-grade phenolic-ester hydrolases (tannases; chlorogenate/phenolic esterases) detail cleavage of galloyl/depsidic and caffeoyl–quinic linkages and summarize fermentation-typical pH/temperature optima, explaining rises in free phenolic acids during plant-food fermentations [114,119].

Engineering strategies can further accelerate isoflavone remodeling. In a soy-beverage context, deploying yeasts with enhanced β-glucosidase capacity increased conversion of isoflavone glycosides to aglycones over conventional fermentations, reinforcing that enzyme dose and stability under acidic, low-temperature conditions are rate-limiting in practice [120].

Finally, legume fermentations illustrate a complementary peptide axis that may act alongside aglycone exposure in cardiometabolic profiles. In jack-bean tempeh, in vitro digestion across intestinal segments yielded absorbed fractions with high ACE-inhibitory activity—jejunum > duodenum > ileum—and peptide-rich hydrolysates showed stronger ACE inhibition than non-absorbed fractions (e.g., approximately 81% vs. approximately 72% inhibition in ileal models), identifying short proline- and hydrophobic-rich sequences as primary contributors [121]. While glycemic endpoints were not measured, these peptide effects co-travel with aglycone-rich matrices and are mechanistically compatible with postprandial modulation in human test meals.

Mechanistically, these aglycone-enriched profiles align with the β-glucosidase toolkit documented for food-grade lactic cultures and yeasts and complement esterase/tannase routes summarized in Section 4.2.1; together they yield smaller, more bioaccessible isoflavone forms within 12–48 h and create a postbiotic milieu (organic acids, EPS, viable LAB) that can plausibly modulate early postprandial glycemia via intestinal and incretin pathways [114,118,119].

#### 4.2.3. Tea Ferments: Tannase/Esterase-Driven Gallic-Acid Release and Organic-Acid Formation

In controlled kombucha prepared with a pure cultured “tea fungus,” fermentation increased total polyphenols by approximately 77.14% and total flavonoids by approximately 69.23%. Quercetin and kaempferol rose by approximately 89.11% and approximately 70.32%, respectively; summed catechins increased by approximately 45.77%, and flavonol glycosides fell by approximately 38.98%. Simulated in vitro bioavailability increased by approximately 29.52% for total polyphenols and by more than sevenfold for flavonoids, together with higher antioxidant readouts (DPPH +43.81%, ABTS +35.08%). Correlation linked these gains positively to epigallocatechin (EGC), epicatechin (EC), epigallocatechin gallate (EGCG), epicatechin gallate (ECG), quercetin, and kaempferol, and negatively with kaempferol-3-glucoside. Mechanistic modeling supported a synergistic route in which organic acids disrupt leaf structure while tannase, polygalacturonidase, and fermentation-derived solvents (acids, alcohols, esters) drive catechin degallation and broader biotransformation [122].

Untargeted FT-ICR-MS mapping in simplified kombucha consortia (*B. bruxellensis*, *H. valbyensis*, *A. indonesiensis*) assigned 506 features to unique formulas (307 retained after replicate filtering) and identified 136 “core metabolites” set. During the open-vessel acidification phase, pH decline from approximately 6.9 to approximately 4.2 and total acidity rose to approximately 20–41 meq/L, with release/formation of gluconic and gallic acids and an expansion of molecular diversity (e.g., approximately 310 detectable masses at day 7 in black-tea kombucha vs. approximately 138 in sugared black tea). In the subsequent closed-vessel phase, oleic acid was consumed and 1,4-β-glucan signatures appeared, indicating cellulose pellicle formation [19].

Across fermented foods more broadly, network analyses show that modest shifts in community composition and co-adaptation histories predictably alter organic-acid and phenolic trajectories, helping explain inoculum-to-inoculum variation in kombucha chemistry [123].

Process comparisons further reinforce enzyme-driven catechin remodeling and its sensory consequences. When tea extracts were sequentially fermented with filamentous fungi (*Trichoderma reesei* or *Aspergillus niger*) followed by LAB, the fungal step hydrolyzed galloylated catechins (key bitterness/astringency contributors), whereas the LAB step decarboxylated subsets of organic and phenolic acids and metabolized saccharides while increasing alcohols and ketones. Sensory evaluation combined with multivariate analysis (Principal Component Analysis and Orthogonal Partial Least Squares-Discriminant Analysis (PCA/OPLS-DA)) clearly separated fermented beverages from their baselines, with floral/fruity gains tracking increases fermented in higher alcohol/ketone and reduced astringency consistent with catechin degallation [124]. In a complementary pickled-tea model, yeast-enhanced fermentation reshaped 198 volatiles and 115 non-volatiles compounds and, by sensory testing, reduced perceived bitterness, astringency, and sourness while enhancing floral notes; odor-activity and regression analyses implicated citronellol and linalool-oxide as contributors and highlighted medium-chain fatty acids as key drivers of the “acidic aroma” signature [125].

Analytically, rapid HPLC methods using silica monolithic columns can resolve eight catechins plus caffeine within approximately 7 min (LODs approximately 0.11–0.29 mg/L; LOQs approximately 0.33–0.87 mg/L; recoveries approximately 94–105%), enabling routine time-resolved quantification of catechin losses (EGCG/ECG) and gallic-acid gains during tea fermentations [126]. Baseline tea chemistry also conditions both sensory and chemical outcomes: In raw Pu-erh teas, taste-addition experiments identified theophylline and rutin as key positive modulators of sweet aftertaste (among 96 annotated taste components), underscoring why starting composition and mouth-residence chemistry matter when interpreting post-fermentation flavor and acceptability [127].

Together, these data delineate a coherent route in tea ferments: Fungal/yeast/lactic tannase and esterase hydrolyze galloyl linkages (e.g., EGCG/ECG → gallic acid + degallated catechins), acetic-acid bacteria expand organic-acid pools (acetic, gluconic) and synthesize cellulose pellicle, and process variables (tea type, inoculum history, fermentation/finishing conditions) shape the exact trajectories. These are primarily analytical and mechanistic observations, but they define specific, measurable catechin to-gallic-acid and sugar-to-organic-acid pathways that should be co-reported with human glycemic and microbiota outcomes when evaluating black- vs. green-tea kombucha and related ferments in clinical settings.

#### 4.2.4. Carbohydrate Remodeling and Viscosity: EPS, BC, and γ-PGA as Proximal Modulators of Starch Digestion

Fermented plant matrices frequently accumulate high-molar-mass microbial polymers—EPS, BC, and γ-PGA—that increase apparent viscosity, water binding, and gelation. These properties can slow starch–enzyme mass transfer and diffusional glucose outflow, providing a purely physical, meal-proximal route to blunting early postprandial glycemic excursions. Below we summarize the main polymer classes and the evidence linking their rheological behavior to digestion rate, noting where support comes from human meal tests (γ-PGA in natto) vs. rheology and in vitro digestion models (EPS/BC).

- EPS from LAB in plant ferments. LAB in vegetable, cereal, and plant-milk fermentations often synthesize high-molar-mass EPS, including dextrans, levans, and heteropolymers. These polymers increase apparent viscosity, water-holding, and gel-like behavior of the matrix, all of which can reduce the effective diffusion of amylases and glucose. Mechanistic and technological studies consistently show that EPS-rich systems become thicker and more elastic as polymer molecular weight and concentration increase (e.g., dextran/levan systems in sucrose-based media and cereal doughs), supporting a direct polymer size → rheology → mass-transfer resistance link [30,128,129].
  In sucrose a model with Gluconobacter albidus TMW 2.1191, levan fractions with higher molecular weight displayed pronounced shear-thinning, increased apparent viscosity, and gel-like viscoelastic behavior (G′ > G″), whereas lower-MW fractions remained mainly viscous [31]. This demonstrates that diffusion-limiting “thickening” capacity scales with polymer size. In kefir and sourdough systems, growth temperature, sucrose availability, and other process variables shift both EPS yield and MW distribution, which in turn alters flow curves and gel properties of the fermented matrix.
  Mechanistically, EPS production is strongly strain- and process-dependent (sucrose availability, pH, temperature), so reporting EPS mass (g/L) and viscosity (mPa·s or flow curves) alongside α-amylase/α-glucosidase assays is essential when linking EPS to glycemic endpoints [38];
- BC in kombucha. In tea fermentations, *Komagataeibacter* spp. synthesize a surface pellicle of kombucha bacterial cellulose (KBC). Structural analyses of green-tea kombucha fermented at approximately 30 °C of KBC harvested on days 7, 14, and 30 showed type-I cellulose by X-ray diffraction (XRD) with crystallinity indices of approximately 90%, approximately 95%, and approximately 91%, respectively; pellicle yields can reach approximately 6.5% under optimized conditions. Functionally, this KBC behaves as a high-surface-area hydrogel: When used as an immobilization matrix for *L. plantarum*, approximately 16.20 log CFU/g were initially adsorbed, approximately 7.98 log CFU/g survived freeze-drying, and approximately 2.94 log CFU/g survived simulated gastric (pH 2.0) plus bile conditions, whereas free cells were undetectable—evidence of strong barrier/retention properties in a digestive-like environment [130].
  When KBC is dispersed or blended, its rheological footprint becomes clearer. Kombucha derived BC incorporated into chitosan solutions yields non-Newtonian, shear-thinning fluids whose viscosity depends on BC loading and temperature; films containing approximately 10% (*w*/*w*) KBC show improved elasticity and slightly higher thermal stability [40]. In nata-de-coco models, chemical modification of BC to carboxymethyl-BC—together with an increased degree of substitution—leads to lower crystallinity and higher solubility and solution viscosity [44], thereby exemplifying the process → polymer structure → rheology relationship.
  Direct in vivo glycemic data with KBC-enriched meals are not yet available, but in vitro starch-digestion models in high-viscosity bread and cereal matrices support the underlying principle: Sourdough and hydrocolloid-enriched breads with higher viscosity and more compact crumb structures show reduced rapidly digestible starch (RDS) fractions and lower predicted glycemic indices compared with texture-matched controls [16]. It is therefore reasonable to hypothesize that KBC, when present as a dispersed nanofibrillar network in the consumed beverage or co-formulated foods, would contribute similarly to slowing starch hydrolysis and glucose diffusion. To test this explicitly, future kombucha trials should report pellicle mass/thickness (or dispersed-fiber content) and simple flow curves for the final product, ideally pairing these with in vitro assays.
- γ-PGA in natto as a viscosity “positive control.” Natto, a *Bacillus*-fermented soy food, provides clinical proof-of-principle that microbial polymers can acutely blunt glycemic excursions. γ-PGA, the sticky, anionic polymer responsible for natto’s characteristic “stringiness”, is present at much higher levels in some formulations than in others. In randomized crossover meal tests in healthy adults, high-γ-PGA natto—delivering several hundred milligrams of γ-PGA per serving— lowered glucose iAUC_0–120_ by approximately 20% vs. white rice and suppressed early-phase glucose and insulin responses compared with low-γ-PGA natto, consistent with a viscosity-mediated slowing of starch digestion and/or glucose diffusion [18]. A separate crossover study that quantified γ-PGA doses (approximately 58 vs. approximately 440 mg per 40 g) similarly found lower early-phase glucose and insulin iAUC with the high-γ-PGA product [22]. Key polymer and process variables to report are summarized in Table 1.
  Outside these meal tests, γ-PGA is a well-characterized anionic hydrocolloid. Work with food-borne *Bacillus* isolates shows robust γ-PGA biosynthesis under fermentation-relevant pH/temperature, yielding very high-molar-mass, water-soluble polymers that build network viscosity and exhibit pronounced shear-thinning; rheological properties are tunable via oxygen transfer, carbon source, and ionic environment [41,42,43]. Applied summaries in nutrition and animal-science contexts note γ-PGA’s mucoadhesive behavior and gastrointestinal stability, implying prolonged luminal residence and thickening of the unstirred water layer over starch surfaces [131]. Materials studies document γ-PGA hydrogels and co-hydrogels with strong water binding, high zero-shear viscosity, and film-forming performance [132].
  Together, these polymer traits provide a direct physical rationale for the human crossover data with high-γ-PGA natto and offer a mechanistic benchmark for interpreting EPS- or bacterial-cellulose-rich plant ferments when polymer dose and rheology are quantified.
- How much do polymers matter in practice? Across fermented plant foods, the extent to which polymers modulate glycemia depends less on their identity (EPS vs. BC vs. γ-PGA) than on physicochemical traits: MW distribution, concentration, conformation, charge, and hydration state. Evidence spans all three polymer classes:
  - For levan from *G. albidus* TMW 2.1191, increasing MW (and, at a given MW, increasing concentration) drives stronger shear-thinning, higher apparent viscosity, and more elastic, gel-like behavior—features that directly increase mass-transfer resistance in model systems [31]. In LAB EPS produced in situ, growth temperature and medium composition shift EPS yield and MW distribution, which in turn changes flow curves and gel properties in fermented dairy and cereal matrices [38,39]. In dough systems, in situ dextran formation by *Weissella* can increase viscosity by several-fold vs. controls, underscoring how polymer build-up alters diffusion and texture at realistic solids levels [133].
  - For BC, both network content and chemical modification tune rheology, as shown in chitosan/BC blends and carboxymethyl-BC solutions [44]. These systems behave as shear-thinning, non-Newtonian fluids whose viscosity sits squarely in the range where in vitro starch digestion rates are reduced.
  - For γ-PGA, bioprocess variables that affect titer and MW can even change broth viscosity during production—properties that translate into digestion-rate effects when γ-PGA is present in the meal, as suggested by the natto crossover data [41,42,43].

Finally, polymer–polymer and polymer–protein interactions can amplify viscosity and network strength. In kefir, for example, adding a polymerized whey–protein/pectin thickening system increases viscosity and densifies microstructure, with evidence of intermolecular aggregation between added pectin and in situ EPS [45]. In bread, sourdough and long-fermentation protocols modify the protein–starch matrix and in vitro starch digestibility in ways that track with measured textural and rheological changes [16].

From a translational perspective, these data collectively support a viscosity-based mechanism whereby microbial polymers—EPS, BC, and γ-PGA—can attenuate early postprandial glycemia. However, only γ-PGA in natto currently has direct human meal-test evidence. For kombucha and other EPS/BC-rich plant ferments, the mechanistic case rests on rheology and in vitro digestion models; future human studies should therefore co-report polymer metrics (yield, MW proxies, viscosity/flow curves) and starch-digestion endpoints (in vitro and postprandial) to strengthen enzyme → polymer → glycemic-response attribution.

#### 4.2.5. Beyond Viscosity: Postbiotic Metabolites and Microbial Biotransformations That Modulate Starch Digestion and Glycemia

Whereas Section 4.2.4 addressed polymer-driven viscosity, here we examine non-polymeric metabolites that act at the enzyme and epithelial levels. This subsection focuses on low-molecular-weight metabolites arising from fermentation (or delivered with fermented foods) and their proximal effects on starch digestion and postprandial glycemia. We emphasize (i) organic acids and SCFAs, (ii) gastric emptying and incretin signaling, and (iii) phenolic and indole derivatives generated by microbial remodeling, and how these link to enzyme inhibition, delivery kinetics, and incretin responses.

- Organic acids and SCFAs—delivery, kinetics, and acute glycemic effects. Across controlled trials and meta-analyses, small organic acids supplied with fermented or acidified foods can attenuate early postprandial glycemia, whereas direct SCFA delivery shows limited acute effects on glucose and insulin in humans. In a 46-study meta-analysis (*n* = 913), vinegar acutely reduced postprandial glucose [standardized mean difference (SMD) −0.53 (95% CI −0.92, −0.14) in impaired glucose tolerance/T2D; −0.27 (−0.54, 0.00) in healthy adults], while acute acetate and acute/chronic propionate showed no significant effects on glucose or insulin [134].
  In an equicarbohydrate randomized crossover in healthy adults, incorporating a vinegar-soaked dried-apple product with a white-rice meal lowered 2 h glucose iAUC by 22.9% vs. control rice; preloading the dried-apple product 30 min before lowered iAUC by 26.3%; soaking the rice in vinegar lowered iAUC by 16.4%. Early insulin iAUC (0–30 min) was 19.7% lower with the preloaded dried-apple condition [28]. These data support a proximal role for luminal acids and gastric kinetics (rather than systemic SCFA per se) in moderating early glycemic excursions.
  By contrast, colonic infusion of physiologic SCFA mixtures (200 mmol/L; acetate-, propionate-, or butyrate-rich) in overweight men (*n* = 12) increased fasting fat oxidation and resting energy expenditure and raised Peptide YY (PYY), with minimal effects on immediate postprandial glycemia during an oral glucose challenge—indicating metabolic signaling orthogonal to acute glucose lowering [135].
  Similarly, in a double-blind randomized crossover in overweight/obese men (*n* = 14), replacing 24 g maltodextrin with 24 g inulin increased early postprandial fat oxidation and plasma acetate and lowered postprandial glucose and insulin (all *p* < 0.05), consistent with fermentation-derived SCFAs shifting substrate use even when glycemic effects are modest [136]. In rodents, 4 weeks of chicory-root supplementation increased peripheral acetate turnover by approximately 25% (*p* = 0.017) without changes in propionate or butyrate turnover, supporting fiber-driven enhancement of systemic acetate availability [137]. These data support a proximal role for luminal acids and gastric kinetics (rather than systemic SCFA per se) in moderating early glycemic excursions.
- Gastric emptying and incretin signaling as proximal levers. Early glycemic excursions are tightly linked to gastric emptying and cephalic–enteric responses. When oro-pharyngeal exposure is bypassed via intragastric feeding, cephalic-phase responses diminish, and gastric emptying is altered. In a randomized crossover with healthy men (*n* = 10), oral soup vs. intragastric infusion produced a larger early insulin response (0–15 min) [169.0 ± 22.1 vs. 124.1 ± 18.8 pmol·L^−1^·15 min; *p* = 0.028], with no difference in blood glucose; gastric emptying was slower with oral exposure [t½ 85.0 ± 2.7 vs. 79.4 ± 3.3 min; *p* = 0.04], supporting sensory–GI crosstalk as a proximal modulator [138].
  Clinical physiology after Roux-en-Y gastric bypass (RYGB) further illustrates how delivery kinetics reshape incretin effects and glycemia. In patients approximately10 weeks post-LRYGB (*n* = 8) vs. lean and obese controls (*n* = 12 each), pouch emptying was accelerated; Glucagon-like peptide-1 (GLP-1), Glucose-dependent insulinotropic polypeptide (GIP), and PYY AUC_60_ were higher in RYGB, with normalized fasting glucose/insulin and good tolerance even to 25 g oral glucose [139].
  For real foods, two randomized, double-blind crossover trials comparing sourdough-fermented pasta with conventional pasta found faster gastric emptying with sourdough (higher paracetamol AUC; shorter gastric emptying (GE) at 30 and 45 min, *p* < 0.05) but no overall differences in postprandial glucose, insulin, or incretins acutely; over 5 days, there were no changes in Oral Glucose Tolerance Test (OGTT) glycemia, with a decrease in fungal α-diversity and lower total fecal SCFAs after sourdough [140]. In parallel, a separate crossover RCT with bread showed person-specific glycemic responses to sourdough vs. white bread linked to microbiome features, underscoring inter-individual variability in delivery–response coupling [5]. Collectively, these data suggest that fermented matrices may modulate gastric and incretin dynamics, but that detectable glycemic benefits are modest and strongly conditioned by baseline physiology and microbiota.
- Microbial remodeling of phenolics—enzyme-level effects with human readouts. Fermentation alters phenolic profiles toward metabolites that can directly inhibit carbohydrate-digesting enzymes and slow starch breakdown. In vitro, green, oolong, and black tea extracts inhibited human salivary α-amylase and mammalian α-glucosidase; black tea was most potent with inhibitory concentration (IC)_50_ = 0.42–0.67 mg tea leaves/mL (α-amylase) and 0.56–0.58 mg/mL (α-glucosidase). In a rice-noodle digestion model, black tea moderately retarded starch digestion, consistent with enzyme-proximal action [26].
  In humans, a crossover RCT in 17 healthy men showed that adding a purple-potato extract (rich in acylated anthocyanins, 152 mg; other phenolics, 140 mg) to a high-starch meal reduced glucose and insulin iAUC (0–120 min) and lowered early glycemia/insulinemia (e.g., glucose at 20 min, *p* = 0.015, and 40 min, *p* = 0.004; insulin at 20 min, *p* = 0.003; 40 min, *p* = 0.004; and 60 min, *p* = 0.005); fibroblast growth factor 19 (FGF-19) rose at 240 min (*p* = 0.001) [141]. Although this intervention did not involve a fermented matrix, it provides proof-of-principle that microbially relevant phenolic profiles can blunt postprandial glycemia via direct enzyme-level and enterohepatic signaling.
  On the microbiome side, urolithin metabotypes A and B (UM-A/UM-B) are now mechanistically grounded: Investigators isolated a new human gut bacterium that produces UM-A/UM-B and established two defined co-cultures that reproduce the UM-A and UM-B pathways during in vitro fermentation—mapping how ellagic-acid/ellagitannin precursors are converted into bioactive urolithins [142]. Emerging human metabotyping work, although not yet specific to fermented plant foods, indicates that individuals with UM-A or UM-B phenotypes differ in urolithin exposure and in selected cardiometabolic readouts after ellagitannin-rich interventions. This supports the concept that baseline microbial capacity for postbiotic production (e.g., urolithins) could stratify responses to polyphenol-rich fermented foods, even though direct glycemic evidence in this context remains scarce.
- Targeted metabolomics aligned to postprandial time courses. Resolving mechanism-to-endpoint pathways requires time-aligned metabolite measurements. Validated targeted LC–MS/MS workflows enable simultaneous quantification of multiple metabolites in plasma with sensitivity suitable for minute-scale sampling—practical for aligning peaks (e.g., acetate or phenolic conjugates) to 0–120 min glycemic profiles after fermented foods [143]. Method-oriented reviews on food processing and polyphenol bioavailability reinforce that matrix structure, acidity, and microbial remodeling jointly determine the metabolite fingerprints that co-travel with postprandial glucose [144]. For fermented plant foods, pairing targeted metabolomics (organic acids, SCFAs, phenolic conjugates, bile-acid species) with standardized glucose/insulin sampling would allow explicit testing of which postbiotic signatures track with glycemic modulation in humans, and in which phenotypes.

Taken together, human data support a modest, acute glycemia-lowering role for luminal organic acids delivered with foods (e.g., vinegars and, by extension, acidic ferments), whereas direct SCFA dosing mainly affects substrate use and energy expenditure with limited immediate impact on glucose. Gastric emptying and incretin signaling emerge as key proximal levers, but trials with sourdough-type products illustrate that changes in motility do not automatically translate into robust glycemic benefits. Fermentation-altered phenolics and derived postbiotics (including urolithins) provide complementary α-amylase/α-glucosidase and enterohepatic mechanisms, with supportive human readouts in non-fermented models and strong preclinical backing. We therefore recommend that future fermented-food trials pair enzyme assays on the actual matrices and targeted metabolomics with standardized postprandial sampling, explicitly distinguishing mechanisms supported by human data from those that remain primarily preclinical.

#### 4.2.6. Analytical Anchors and Minimal Reporting Set: Enzyme → Metabolite → Endpoint

Building on Table 1 (polymer metrics), this subsection details non-polymeric analytes and design items required for transparent attribution. Here we outline analytical anchors and a minimal reporting set to connect enzyme-level mechanisms to metabolite dynamics and clinical endpoints. We specify what to quantify (analytes, matrices, time points), how to quantify it (HPLC/GC/LC–MS methods, derivatization, internal standards), and how to report trials (checklists) so that enzyme → metabolite → endpoint links are reproducible.

Reliable mechanism-to-endpoint inferences require validated assays on the actual matrices served (and, when relevant, plasma/urine) plus time-aligned sampling against postprandial curves. For fermented plant foods, core targets are as follows: (i) organic acids, residual sugars, and ethanol by HPLC/GC; (ii) phenolics (e.g., isoflavones, phenolic acids) by targeted LC–MS/MS; (iii) polymer surrogates that shape transport (BC mass/thickness; EPS yield and rheology); and (iv) plasma panels that can be synchronized to 0–120 min glycemic readouts. Key analytical anchors and reporting items are summarized in Table 2.

- Matrix compositional anchors. As an example of what to report, a SCOBY-driven fermented milk drink quantified by HPLC contained lactose at approximately 4.25 g/100 g in the lactose version (glucose approximately 2.26 g/100 g in the lactose-free variant) and lactic acid approximately 0.68 g/100 g—numbers that tie pH/titratable acidity to concrete acid loads likely to influence gastric kinetics and enzyme activity. Reporting these values alongside pH and titratable acidity provides the acid base for mechanism mapping [46].
- Targeted phenolic/isoflavone methods. For soy-based ferments and plant milks, validated Liquid Chromatography–Electrospray Ionization–Mass Spectrometry (LC–ESI–MS) workflows resolve aglycones and glycosides (e.g., genistein/daidzein and genistin/daidzin), using extracted-ion chromatograms and characteristic fragmentation; this enables absolute or internal-standard-normalized quantification and direct matrix → metabolite attribution [48]. Untargeted/targeted LC–MS metabolomics in fermented soy whey illustrates how processing shifts organic acids, amino acids, and small phenolics, and why method validation (linearity, accuracy/precision, recovery) should be explicitly documented for any analytes later linked to glycemic endpoints.
- Plasma panels and handling. To time-lock metabolites with glucose/insulin, LC–MS/MS panels validated for aqueous acids/oxo-acids (e.g., lactate, pyruvate, α-ketoacids, ketone bodies) can be deployed with appropriate derivatization (e.g., 4-bromo-N-methylbenzylamine or O-benzylhydroxylamine) and full performance characteristics (matrix effects, linearity, precision, recovery, stability). Critically, pre-analytical handling (rapid processing, −80 °C storage) must be specified because some targets (e.g., acetoacetate) are unstable—details that directly affect metabolite → endpoint alignment [47].
- Polymer surrogates that modulate transport. KBC yield and structure can be captured by simple gravimetry/thickness and complemented with basic rheology when dispersed: KBC film yields of approximately 8.3 ± 2.6 g/L and thicknesses on the order of 0.04–0.46 mm have been reported, and chitosan/KBC composites illustrate how small changes in polymer loading reshape viscosity (e.g., approximately 14 → 107 mPa·s as chitosan increases from 0.5 → 1.5%). Those measurements—mass, thickness, viscosity curves—are the minimal anchors to connect polymer content to diffusion-limited starch hydrolysis [49].
- EPS readouts. Because in situ EPS output is process-sensitive and can shift molecular-weight distributions without obvious changes in total grams, studies should report both an EPS mass proxy (e.g., phenol–sulfuric carbohydrate assay) and techno-functionals (apparent viscosity/flow curves). In dairy systems, (i) kefir formulated with a polymerized whey-protein/pectin thickening system showed higher viscosity and a denser microstructure than controls, with spectroscopic evidence of interactions between added pectin and in situ EPS—underscoring why flow curves should accompany EPS quantification [45]. (ii) Purified kefiran from kefir grains exhibits high zero-shear viscosity, strong water binding, and film/mucoadhesive behavior under food-relevant pH/ionic strength; these solution properties provide an upper-bound benchmark for in situ EPS contributions and justify reporting shear-thinning and apparent viscosity on the actual matrices [50].

## 5. Mechanistic Insights Linking Fermentation to Glycemic Control

**Figure 1:**
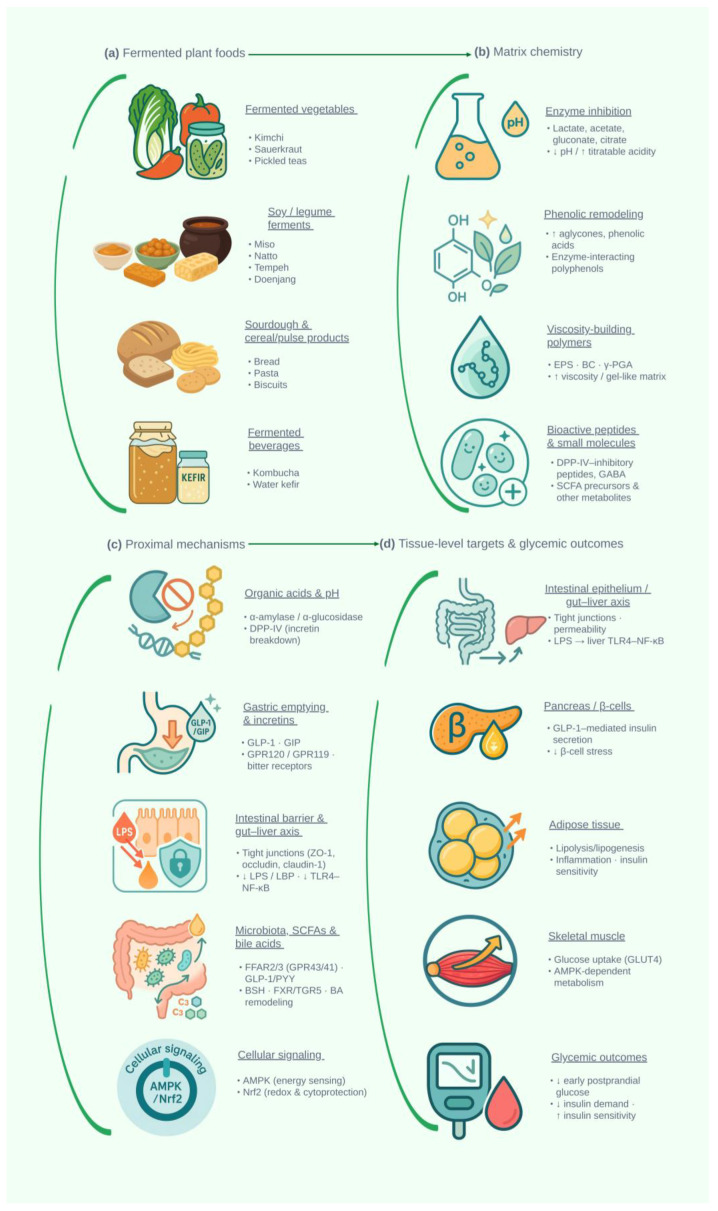
Integrative mechanistic map linking fermented plant foods to glycemic control. The schematic aligns matrix-derived features (organic acids, phenolic remodeling, viscosity-building polymers such as EPS, BC, and γ-PGA) with proximal mechanisms (α-amylase/α-glucosidase inhibition, gastric emptying/incretin signaling, intestinal-barrier reinforcement, and microbiota-dependent SCFA/bile-acid signaling) and tissue-level targets (intestinal epithelium/gut–liver axis, pancreas, adipose tissue, and muscle). Arrows indicate hypothesized directions of effect; dashed lines denote context-dependent or indirect routes. Abbreviations: EPS, exopolysaccharides; BC, bacterial cellulose; γ-PGA, Poly-γ-glutamic acid; DPP-IV, dipeptidyl peptidase-IV; GABA, γ-aminobutyric acid; SCFA, short-chain fatty acids; LPS, Lipopolysaccharide; TLR4, Toll-like receptor 4; NF-κB, Nuclear Factor-kappa B; GLP-1, Glucagon-like peptide-1; GIP, Glucose-dependent insulinotropic polypeptide; LBP, Lipopolysaccharide-binding protein; FFAR2/3, free fatty acid receptors 2/3; PYY, Peptide YY; BSH, bile-salt hydrolases; FXR/TGR5, bile-acid receptors; AMPK, AMP-activated protein kinase; Nrf2, nuclear factor erythroid 2-related factor 2.

An integrative schematic (Figure 1) summarizes matrix chemistry, proximal mechanisms (enzyme inhibition, gastric emptying/incretin signaling, and microbiota-dependent SCFA/bile-acid signaling), and tissue-level targets (intestinal epithelium, liver, adipose tissue, pancreas, and muscle) that may connect fermented plant foods to glycemic control. In line with the methodological framework of this review, the pathways depicted should be interpreted primarily as mechanistic hypotheses, with varying degrees of support from human vs. preclinical data: Some (e.g., viscosity-mediated attenuation of early postprandial glycemia) have direct human proof-of-principle, whereas others (e.g., AMPK/Nrf2 activation, bile-acid signaling) are currently supported mainly by rodent models and in vitro work.

### 5.1. Intestinal Barrier and Gut–Liver Axis

Disruption of epithelial tight junctions increases endotoxin translocation (circulating Lipopolysaccharide (LPS)), activates hepatic Toll-like receptor 4 (TLR4) and Nuclear Factor-kappa B (NF-κB) signaling, and worsens insulin resistance; conversely, fermented-food matrices and their derivatives recurrently tighten the barrier and lower endotoxemia in preclinical models, with concordant improvements in liver inflammation and metabolism [145,146]. In fermented-tea models, barrier restoration is quantifiable: In Dextran Sodium Sulfate (DSS) colitis, a yellow-tea intervention reduced the Disease Activity Index (DAI) from 7.00 ± 0.58 to 2.25 ± 0.50 by day 7 (*p* < 0.001), restored ZO-1/occludin, and lowered serum LPS together with IL-1β/IL-6/TNF-α—a profile consistent with reduced translocation of microbial products to the liver [147]. In a complementary high-fat-diet (HFD) model using Tibetan tea fermented with *Bacillus licheniformis*, intestinal mucin and junctional proteins increased (MUC1 2.09×; MUC2 2.87×; ↑ claudin-1, occludin, ZO-1/ZO-2) while colonic TNF-α and p-NF-κB p65 declined, again pointing to a barrier-centric route for dampening gut–liver inflammatory signaling [148].

Strain- and matrix-level evidence extends beyond simple colitis models. In DSS colitis, combining *Bacillus natto* JLCC513 with ginseng soluble dietary fiber attenuated clinical severity and histologic damage while reinforcing tight-junction markers and down-modulating the TLR4/MyD88/NF-κB axis; pro-inflammatory cytokines fell, and gut-microbiota composition shifted toward a less inflammatory profile—direct support for a barrier-first route to downstream hepatic/metabolic benefits [149]. In production animals, fermenting palm-kernel cake with *Lactobacillus plantarum* LY19 + *Bacillus natto* NDI and feeding it to broilers improved intestinal morphology, increased Claudin-1 expression, and raised total antioxidant capacity vs. controls; SCFA levels and global caecal diversity were largely unchanged, but liver metabolomic profiles shifted toward amino-/carbohydrate-metabolism pathways—illustrating how fermented-matrix inputs can tighten the barrier yet modulate liver biology even when luminal SCFAs do not rise [150].

At the molecular level, theaflavin-3,3′-digallate (TF3)—a black-tea fermentation-derived polyphenol—strengthened intestinal tight junctions and attenuated hepatic oxidative–inflammatory signaling while preventing alcoholic liver injury in mice, placing a specific fermented-tea constituent on the intestine → liver causal path [151]. Complementing this, *Lactobacillus plantarum* ZJUFB2 in HFD-fed mice (16 weeks, approximately 10^9^ CFU/day) improved glucose tolerance and insulin resistance, lowered circulating LPS and Lipopolysaccharide-binding protein (LBP), increased fecal SCFAs (notably acetate, i-butyrate, i-valerate; ↑ total SCFAs), and modulated bile-acid metabolism (↑ hepatic total bile acids alongside enrichment of bile-salt hydrolases (BSH)-containing taxa)—supporting an endotoxemia-to-insulin-sensitivity link [152].

Collectively, these data position intestinal barrier integrity as a proximal node linking fermented-food matrices to reduced endotoxemia (LPS/LBP), dampened hepatic TLR4–MyD88–NF-κB signaling, and improved insulin sensitivity. However, it is important to note that this evidence is predominantly preclinical; most human fermented-food trials to date have not measured permeability markers or LPS/LBP in parallel with glycemic outcomes. As a result, improved glucose or lipid profiles in humans can be consistent with barrier-mediated mechanisms but cannot yet be firmly attributed to them.

To strengthen mechanism-to-endpoint inference and narrow this preclinical gap, future studies should co-report tight-junction proteins (ZO-1, occludin, claudin-1), functional permeability (e.g., fluorescein isothiocyanate (FITC)-dextran, diamine oxidase (DAO), D-lactate), endotoxemia markers (LPS, LBP, sCD14), and hepatic biochemistry/signaling (alanine transaminase (ALT)/aspartate transaminase (AST), TLR4–NF-κB), alongside standardized matrix chemistry (pH/titratable acidity, organic acids, residual sugars/ethanol, key phenolics), dose, and sampling windows. With barrier function stabilized as a plausible node, the next question is how fermented matrices shape postprandial incretin biology—GLP-1 and GIP secretion and DPP-IV activity—which we address in Section 5.2.

### 5.2. Incretin Signaling (GLP-1, GIP) and DPP-IV (Bioactive Peptides)

Fermented plant matrices can interface with incretin biology via two complementary routes: (i) stimulating GLP-1/GIP secretion from enteroendocrine cells through nutrient-sensing G protein-coupled receptors (GPCRs) (e.g., FFAR2/3 for SCFAs; GPR120/GPR119 and bitter-taste receptors engaged by fermentation-remodeled polyphenols and small acids), and (ii) prolonging incretin bioactivity by attenuating DPP-IV-mediated cleavage via food-derived peptides and select polyphenols. Recent syntheses consolidate these mechanisms across plant foods and beverages—including fermented teas and legume/soy systems—providing a clear rationale to test incretin endpoints in fermented-food trials [27]. At present, however, very few human studies with fermented plant foods have measured active/total GLP-1, GIP, or DPP-IV activity, so most evidence for this axis remains preclinical or inferred from non-fermented peptide preparations.

#### 5.2.1. Fermented Teas: GLP-1 Secretion

In dark/fermented-tea models, teabrownins increase circulating GLP-1 and improve postprandial control in mice; in enteroendocrine secretin tumor cell-1 (STC-1) cells, they stimulate GLP-1 release—consistent with GPCR-based signaling from fermentation-derived polyphenols [153]. Complementary mechanistic work identifies teadenol A, a fermented-tea metabolite, as a GPR120 agonist that augments GLP-1 secretion, supporting a receptor-level pathway from tea fermentation to incretin signaling [154].

These findings suggest that oxidized/dark-tea fermentations can generate metabolites with incretin-active potential. Nevertheless, human incretin readouts in fermented-tea or kombucha trials are essentially absent; most clinical work has focused on glucose, insulin, and lipids, without parallel GLP-1/GIP or DPP-IV measurement and with heterogeneous tea bases (black vs. green) and background diets (Section 3.1.3). Thus, GLP-1 stimulation by fermented teas should currently be viewed as a mechanistic hypothesis with strong preclinical support but limited human confirmation. Future trials should prioritize standardized active/total GLP-1 preanalytics [27] and integrate incretin endpoints into glycemic test meals.

#### 5.2.2. Legume/Soy Fermentations: DPP-IV Inhibition by Peptides

Proteolysis during legume/soy fermentation liberates short, proline- and hydrophobic-rich peptides with DPP-IV inhibitory activity—one of the canonical antidiabetic mechanisms ascribed to fermented soybean foods (miso, doenjang, tempeh). Contemporary reviews synthesize this peptide route and its analytical hallmarks across soybean matrices, positioning fermented-soy products as plausible sources of GLP-1-preserving activity in mixed meals [155].

Quantitatively, food-grade plant protein hydrolysates bracket realistic potency ranges: In a head-to-head evaluation, soybean and pea protein hydrolysates showed DPP-IV IC_50_ values of approximately 1.15 and approximately 1.33 mg/mL, respectively, with enriched proline and hydrophobic residues in active sequences—parameters that map well onto peptides released during fermentation [156].

Fermentation itself can heighten functional readouts and generate transport-competent peptides. In green lentils, 72 h solid-state fermentation with *L. plantarum* increased apical DPP-IV inhibition in a Caco-2 intestinal model to 37.3% (vs. unfermented), with detectable basolateral activity (7.9% inhibition at 500 mg/mL) and identification of peptide fractions capable of transepithelial passage—demonstrating both enhanced inhibition and intestinal permeability of fermentation-derived peptides [157].

Together, these data support a peptide-centric mechanism whereby legume/soy fermentations supply DPP-IV-inhibitory oligopeptides at physiologically relevant potencies. However, human evidence linking these peptides to incretin profiles is still missing; to our knowledge, no trial has yet co-measured plasma DPP-IV activity and active GLP-1 after fermented-soy meals with well-characterized peptide profiles. Bridging this gap—by integrating targeted peptidomics with incretin and glycemic endpoints—will be critical to validate this mechanism in vivo.

### 5.3. AMPK and Nrf2: Redox/Inflammatory vs. Ion ↔ Insulin Sensitivity

Fermented plant matrices interface with cellular energy/redox control chiefly through AMPK and the Nrf2 antioxidant pathway—two hubs that improve insulin sensitivity by curbing lipogenesis, oxidative stress, and inflammatory signaling. In oxidized/fermented tea models, a single oral dose of theaflavin-rich fraction increased whole-body energy expenditure and significantly elevated phosphorylated AMPKα in skeletal muscle within 2–5 h, aligning tea fermentation products with acute AMPK activation in vivo [158].

Soy-based ferments provide converging, diet-relevant evidence. In high-fat-diet mice, fermented soybean paste (doenjang) reduced hepatic triglycerides and downregulated lipogenic genes while activating AMPK—documented by increased p-AMPK and suppression of SREBP-1c/FAS—supporting an AMPK-mediated anti-steatotic effect traceable to the fermented matrix [33]. Extending mechanistic scope, a recent mouse study with traditionally prepared doenjang reported up-regulation of hepatic Nrf2 (Nfe2l2) and HO-1, together with higher p-AMPK/AMPK, lower malondialdehyde, and improved serum lipids vs. controls, placing long-fermented soy on the Nrf2 ↔ AMPK axis that links antioxidant defense to improved metabolic readouts [159].

Tea-based ferments can engage both nodes simultaneously: In an ethanol-injury model, ginseng-berry kombucha attenuated hepatic damage while activating AMPK and Nrf2/HO-1 and normalizing oxidative/inflammatory markers, indicating that kombucha-style acid/polyphenol milieus can transmit redox-metabolic benefits along the gut–liver axis [160]. More broadly, systematic appraisals of kombucha report recurring references to Nrf2-linked cytoprotection in preclinical work, reinforcing the plausibility of this pathway in fermented-tea contexts even as human evidence remains sparse [78].

From a translational standpoint, it is crucial to stress that almost all AMPK/Nrf2 data for fermented plant foods derive from rodent models or cell culture. Human RCTs with fermented vegetables, soy, or kombucha have not yet incorporated p-AMPK or Nrf2 readouts in accessible tissues, nor have they systematically linked changes in these pathways to improvements in insulin sensitivity or glycemic control. As a result, AMPK/Nrf2 should currently be regarded as a cross-cutting mechanistic node (P5 in Figure 1) that is biologically plausible and well-grounded preclinically, but still hypothetical in humans.

When AMPK/Nrf2 is posited as a causal route, human interventions should therefore pair glycemic endpoints with fasting/postprandial p-AMPK surrogates in accessible tissues (e.g., PBMC phospho-signatures), circulating and liver injury markers, and validated Nrf2 targets (HO-1/NQO1) in ex vivo assays—while co-reporting matrix chemistry (organic acids, residual sugars/ethanol, hallmark polyphenols) to attribute effects to specific fermented-food components. (See Section 4.2.5 and Section 4.2.6 for analytical anchors). Only with this level of phenotyping can we determine whether the robust AMPK/Nrf2 responses seen in animals translate into clinically relevant improvements in humans.

### 5.4. Microbiota Crosstalk: SCFA Receptors and Bile-Acid Signaling

Microbial SCFAs signal through free-fatty-acid receptors FFAR2/GPR43 and FFAR3/GPR41 on enteroendocrine L-cells, adipose tissue, and immune cells, modulating GLP-1/PYY secretion, insulin sensitivity, and inflammation. Canonical work shows that SCFAs stimulate GLP-1 release via FFAR2, and integrative reviews link SCFA–FFAR signaling to appetite and glucose control [29].

Bile acids (BAs) form a parallel endocrine axis: Activation of TGR5 on L-cells promotes GLP-1 secretion, while the nuclear receptor FXR controls BA synthesis/transport and intersects with glucose–lipid pathways; experimental work and reviews map these control points as relevant to glycemic regulation [161]. Pharmacological manipulation of the BA pool with bile-acid sequestrants has been shown to raise circulating GLP-1 in humans in a TGR5-dependent manner [162].

Where fermented plant foods slot in: Many LAB typical of fermented vegetables/soy/teas encode BSHs that deconjugate bile acids and—recently shown—can also form amine-conjugated bile acids; these activities reshape the intestinal BA pool and modulate FXR/TGR5 tone. Comparative genomics and reviews document a widespread, niche-tuned BSH repertoire across *Lactobacillus lineages* with metabolic consequences [163,164,165].

Polyphenol-rich fermented teas can also remodel BA metabolism upstream. In a *Nature Communications* study, teabrownins (a hallmark polymeric pigment of Pu-erh/dark tea) lowered serum cholesterol while suppressing BSH-positive taxa, increasing ileal conjugated BAs (e.g., tauro-conjugates), down-modulating intestinal FXR–FGF15 signaling, and up-regulating hepatic CYP7A1 with greater fecal BA loss—together consistent with a gut–liver BA feedback that favors GLP-1-permissive signaling and cholesterol disposal [166].

Together, SCFA–FFAR and BA–FXR/TGR5 axes provide plausible endocrine routes through which fermented plant foods—via microbial BSH activity and tea-derived polyphenols—can influence incretin biology and glycemic control. At the same time, direct human evidence that plant-based fermented foods modulate these axes sufficiently to change incretin profiles or glycemia is still limited. Most clinical kombucha and fermented-vegetable/soy trials have measured microbiota composition and standard metabolic markers but not BA species, SCFA kinetics, or GPCR signaling. As underscored in Section 4.1.5 and Section 4.2.5, baseline microbiota composition and function (e.g., SCFA production capacity, BSH activity, urolithin metabotype) are likely to shape individual responses, supporting a personalized nutrition perspective that will need to be tested explicitly (see Section 7).

Accordingly, future trials should co-report BA profiles (conjugated/unconjugated), GLP-1/PYY, and relevant GPCR targets, alongside fecal and circulating SCFAs and detailed product chemistry and diet records. This will allow us to determine when fermented plant foods act mainly as delivery vehicles for acids and polyphenols, and when they genuinely reprogram SCFA and BA signaling via the microbiota, with consequent effects on glycemic control.

## 6. Evidence from Preclinical and Clinical Studies

### 6.1. In Vitro and Animal Models by Matrix

#### 6.1.1. Kimchi and Other Fermented Vegetables: In Vitro and Animal Models

Strains isolated from fermented vegetables (kimchi and related lactic ferments) repeatedly show probiotic hallmarks—acid/bile tolerance and pathogen antagonism—together with enzyme-level effects relevant to glycemic control; across in vitro screens, Lactiplantibacillus/Lactobacillus isolates inhibit digestive carbohydrases (α-amylase/α-glucosidase) and dampen inflammatory readouts in epithelial/immune co-cultures, while extracts/fractions from vegetable ferments likewise display α-glucosidase inhibition in food-relevant models, consistent with fermentation-remodeled phenolics and small organic acids [167,168,169,170,171,172].

- Colitis and barrier-first models (gut–liver axis). In DSS colitis, kimchi-derived *L. mesenteroides* DRC1506 or a freeze-dried kimchi preparation attenuated disease severity and barrier damage. With 3% DSS and daily gavage of 1 × 10^9^ CFU for 21 days, DAI at day 20 fell from approximately 4.7 in DSS controls to approximately 1.6–1.8 with kimchi or DRC1506, and body weight trajectories improved (final weight approximately 113–115% of baseline with kimchi/DRC1506 vs. approximately 109% with DSS). Colon length shortening was also mitigated (median approximately 7.7–8.0 cm vs. approximately 6.5 cm in DSS), while TNF-α decreased and IL-10 increased in serum/colon, and ZO-1/occludin protein levels were restored (*n* = 6–8 per endpoint; ANOVA *p* < 0.05) [167]. These effects support a barrier-first chain—epithelial sealing → reduced endotoxemia → improved inflammatory tone—that generalizes to vegetable-ferment matrices. A complementary kimchi-beverage study in DSS mice reported the same directional pattern (lower DAI, less colon shortening, ↓IL-6/↓TNF-α, ↑ZO-1/occludin), reinforcing external validity across kimchi formats [168].
- Diet-induced obesity and glycemic readouts (rodent). In HFD models, kimchi-style ferments or kimchi-derived LAB improve glucose handling alongside adiposity and hepatic lipid metabolism. In HFD-fed mice, oral *Lactobacillus sakei* OK67 (kimchi isolate; 12 weeks) significantly improved oral-glucose-tolerance (lower OGTT AUC), reduced adiposity and hepatic triglycerides, and down-modulated intestinal inflammatory markers, consistent with an anti-obesity effect that extends to glycemic control (*p* < 0.05) [169]. Separately, HFD mice consuming kimchi supplemented with a citrus concentrate showed reduced fasting glucose and insulin with lower HOMA-IR vs. HFD controls, alongside favorable changes in adiposity and hepatic lipogenic gene expression, consistent with improved insulin sensitivity (*p* < 0.05) [170]. In additional HFD models, a kimchi-isolated *Lactobacillus plantarum* (Ln4) reduced weight gain and fat mass and improved oral glucose tolerance, while a separate kimchi-derived *L. fermentum* (SMFM2017-NK4) prevented diet-induced obesity and improved lipid profiles; although glycemic endpoints were not primary in the latter, the metabolic directionality is concordant with the Ln4 findings [171].
- Microbiota remodeling as a co-driver. In overweight adults, a randomized, double-blind, placebo-controlled trial (*n* = 55; 60 g/day freeze-dried kimchi for 12 weeks) reported a 2.6% decrease in body fat in the kimchi group vs. a 4.7% increase with placebo. 16S profiling showed increased *A. muciniphila* and reduced Proteobacteria with kimchi, consistent with a shift toward a metabolically favorable community [172]. These human data complement rodent studies and align with the SCFA–FFAR and BA–FXR/TGR5 axes outlined in Section 5. However, most direct glycemic outcomes for kimchi in humans come from small, short-term trials (Section 3.1.1), so the animal models in this subsection should be interpreted primarily as mechanistic support rather than definitive proof of clinical efficacy.

#### 6.1.2. Soy Ferments (Tempeh/Miso/Natto; Fermented Soybean Meal)

Across rodent models, soy ferments improve glucose handling and insulin resistance while attenuating hepatic injury. In HFD rats, daily oral administration of fermented soybean meal produced with *Lactobacillus plantarum* FPS2520 and *Bacillus subtilis* N1 for 6 weeks significantly lowered fasting glucose, fasting insulin, and HOMA-IR and reduced OGTT AUC vs. HFD controls; liver injury markers (AST/ALT) also fell, consistent with improved glycemic control and hepatic status under a fermented-soy background [173]. In HFD-fed mice, supplementation with cheonggukjang (a short-ripened fermented soybean paste) led to significantly lower fasting glucose and insulin than HFD controls and improved hepatic steatosis, indicating anti-obesogenic effects that extend to glycemic endpoints [174]. Mechanistically, the fermented-soy intervention in rats above used a defined two-stage fermentation (first *B. subtilis*, then *L. plantarum*), and authors discuss synergy between microbial metabolites and isoflavone remodeling, aligning with enzyme-level routes (e.g., DPP-IV/α-glucosidase inhibition) prioritized earlier in Section 5. Nonetheless, these benefits have been demonstrated under obesogenic, high-fat contexts and relatively high doses in rodents; human data on tempeh/miso/natto summarized in Section 6.2.2 generally show more modest effects on glycemic endpoints.

#### 6.1.3. Kombucha

In diet-induced obese mice with NAFLD features, Xu et al. [175] reported that daily kombucha supplementation improved oral glucose tolerance (lower OGTT AUC), reduced fasting hyperinsulinemia, and ameliorated hepatic steatosis and collagen deposition. Mechanistically, kombucha normalized insulin signaling (restored insulin-stimulated Akt phosphorylation), down-modulated hepatic bile-acid receptor pathways (TGR5/FXR) and shifted gut microbiota toward health-associated taxa (e.g., enrichment of *Akkermansia*), aligning liver histology and signaling with better systemic glucose handling [175]. These findings echo earlier kombucha studies summarized above: Cardoso et al. [77] showed improvements in glucose metabolism and liver fat under a HFFD background, and Moreira et al. [76] reported better glucose tolerance with mitigation of hepatic steatosis—together consolidating kombucha’s preclinical signal on glycemic control and liver health.

#### 6.1.4. Kefir (Water/Soy)

In streptozotocin–nicotinamide (STZ/NA) diabetic rats, a mixed goat–soy kefir increased endothelial NO bioavailability via Endothelial Nitric Oxide Synthase (eNOS) upregulation, together with anti-inflammatory signals—placing kefir matrices on a vascular–metabolic route [176]. In HFFD models, soy-milk kefir inhibited α-amylase/lipase in vitro and improved metabolic readouts in vivo, consistent with enzyme-level attenuation of postprandial excursions [32]. Complementing these data, a water-kefir grain-derived *L. paracasei* strain strengthened intestinal tight-junction markers and reshaped the gut microbiota in diabetic mice, aligning barrier repair with metabolic improvements—a barrier → metabolism route relevant to tibicos/water-kefir matrices [177]. As with kombucha, however, most of this evidence remains preclinical, and extrapolation to non-dairy kefirs consumed by humans should be made cautiously until dedicated trials are available.

#### 6.1.5. Sourdough

Sourdough fermentation reorganizes the cereal starch–protein matrix and enriches dough with organic acids, EPS (including β-glucans and dextrans), phenolics, and peptides. Together, these changes modulate starch gelatinization, enzyme accessibility and colonic fermentability, making sourdough an archetype of “postbiotic” bakery matrices with potential for glycemic modulation. Recent syntheses highlight that sourdough can increase resistant starch (RS) and slowly digestible starch (SDS), lower RDS, and thereby reduce the glycemic impact of cereal products, though effects are highly context-dependent [23].

- In vitro digestibility and predicted GI. Controlled breadmaking studies show that sourdough per se does not guarantee a lower glycemic response; benefits emerge when fermentation type, time, and substrate are optimized. In model wheat and whole-wheat breads, Demirkesen-Bicak et al. [16] systematically varied flour type, fermentation temperature (25 vs. 30 °C), and sourdough process (Type I vs. II). Estimated GI (eGI), derived from Englyst starch fractions, fell by almost 30% in the most acidified, Type II whole-wheat sourdough fermented at 30 °C, whereas other combinations produced modest or negligible changes despite similar organic-acid levels.
  Composite cereal products confirm that process parameters and microstructure are key. In Pinsa Romana, an elongated pizza-bread, a long-fermented biga containing sourdough (48 h at 16 °C) produced the lowest predicted GI and reduced in vitro glycemic response compared with shorter fermentations or yeast-only doughs, while simultaneously increasing GABA, total peptides, and essential amino acids—consistent with a protein-enriched, more SDS matrix [178]. In semolina pasta reformulated with red-lentil protein isolate, sourdough fermentation of semolina regrinds lowered the starch hydrolysis index and predicted GI vs. non-fermented controls, despite only modest shifts in total RS, pointing to subtle rearrangements of the protein–starch network and amylopectin retrogradation as levers on digestibility [179].
  Beyond baked bread and pasta, leavening agents also modulate digestibility in steamed cereal matrices. In Chinese steamed bread, replacing baker’s yeast with regional sourdoughs altered crumb porosity and the partitioning of RDS vs. SDS, underscoring the central role of sourdough microflora and fermentation history in structuring starch accessibility—even without crust formation [180].
  Not all “functionalized” sourdough products show clear glycemic benefits. When olive-oil mill wastewater was incorporated into sourdough bread as a phenolic-rich ingredient, total phenolics and antioxidant capacity increased several-fold, but in vitro starch digestibility and predicted postprandial glycemia were essentially unchanged relative to the control bread [181]. Likewise, in whole meal breads, Verdonck et al. [182] reported that sourdough starters and fermentation regimes modestly increased RS but did not substantially alter the rate constant or final extent of starch digestion vs. yeast-fermented controls—suggesting “ceiling effects” when the base matrix is already fiber-rich. Together, these data illustrate that flour type, fermentation mode (Type I vs. II), temperature, and fermentation time must be tuned to translate sourdough’s biochemical changes into meaningful reductions in starch digestibility and eGI.
- In vivo models integrating glycemia, lipids and microbiota. The most complete preclinical dataset comes from a mouse feeding study that directly compared yeast-leavened white bread with sourdough bread. Kwon et al. [183] fed mice diets containing yeast-fermented white bread, white bread with 40% sourdough, or unbaked sourdough for 11 weeks. Despite similar carbohydrate content and higher feed intake/body weight in the sourdough-bread group, blood glucose excursions were blunted and GI, calculated from glucose AUC, was significantly lower in the sourdough-bread group than in the yeast-bread group. This low-GI phenotype coincided with lower plasma total cholesterol and triglycerides, a more favorable LDL:HDL ratio, reduced pro-inflammatory cytokines (TNF-α, IL-6), and enrichment of *Akkermansia, Bifidobacterium,* and *Lactobacillus* in the gut microbiota. These changes are compatible with mechanistic data showing that sourdough fermentation increases SDS, EPS, and organic acids and enhances colonic SCFA production, which together can modulate glucose–lipid–inflammatory axes [23].
- Postbiotic fibers, β-glucans, and phytate degradation. Defined-culture studies show that sourdough can be engineered as a carrier of specific polysaccharides with prebiotic and chemopreventive potential. Schlörmann et al. [184] used β-glucan-producing *Levilactobacillus brevis* and *Pediococcus claussenii* as sourdough starters in wheat and rye. After simulated gastrointestinal digestion and colonic fermentation, breads fermented with β-glucan-positive strains yielded higher SCFA production and stronger antiproliferative effects in colon cell assays than breads made with isogenic β-glucan-deficient mutants, indicating an added value of in situ EPS enrichment beyond simple acidification. In parallel, Fekri et al. [185] showed that phytate-degrading probiotic LAB and yeasts from traditional sourdoughs reduced phytic acid, increased mineral bioaccessibility, boosted exopolysaccharide and phenolic contents, and altered in vitro starch digestibility in whole-wheat breads, placing sourdough at the intersection of mineral metabolism, antioxidant defenses, and glycemic modulation.
- Bioactive peptides and enzyme-level actions. Beyond matrix and fiber effects, sourdough breads can deliver peptide-level activities relevant to cardio-metabolic risk. In a recent study, Bartos et al. [34] selected gliadin-degrading *Lactobacillus* strains for laboratory sourdoughs and produced wheat and wheat–rye breads whose in vitro digests showed pronounced ACE inhibition across a range of simulated digestive conditions, whereas α-amylase inhibition was less consistent. These findings position sourdough breads not only as potential low-GI carriers but also as vehicles for cardio-metabolic postbiotics (ACE-inhibitory peptides, β-glucans, EPS) whose effects extend beyond glycemic excursions.

Overall, the sourdough preclinical literature supports a coherent chain of mechanisms: Acidification, organic-acid production, and EPS formation, restructuring of the starch–protein–fiber matrix, and liberation of peptides and phenolics can synergistically lower GI and improve lipid and inflammatory profiles. At the same time, the magnitude—and sometimes even the presence—of glycemic benefits is highly formulation-dependent, varying with flour type (refined vs. whole, gluten-free pseudo-cereals), sourdough process (Type I vs. II, defined vs. spontaneous starters, fresh vs. freeze-dried), fermentation time/temperature, and co-ingredients. This context dependence is crucial for interpreting human intervention trials and head-to-head comparisons in Section 6.2 and Section 6.3, where ostensibly similar “sourdough” products may differ sharply in their capacity to translate these mechanistic advantages into clinically relevant improvements in postprandial glycemia.

### 6.2. Human Interventions by Matrix and Endpoint

#### 6.2.1. Kombucha and Kefir

Human intervention data are still sparse for tea- and kefir-based ferments, but two kombucha trials and one kefir trial begin to outline their metabolic profile. In an acute randomized crossover GI study (*n* = 11), a standardized high-GI rice meal was consumed with soda water, diet soft drink, or an unpasteurized live kombucha; while GI and insulin index (II) were virtually identical with soda water and diet soft drink (GI approximately 84–86; II approximately 81–85), kombucha reduced both indices to approximately 68 and approximately 70, respectively (*p* = 0.041 vs. soda water), indicating a statistically significant attenuation of acute postprandial glucose and insulin responses in this small pilot [72]. Complementing this acute signal in healthy volunteers, Mendelson et al. [71] performed a double-blind randomized crossover pilot in adults with T2D (*n* = 12), assigning participants to 240 mL/day kombucha or a sensory-matched placebo for 4 weeks, with an 8-week washout. Kombucha lowered mean fasting blood glucose from 164 to 116 mg/dL over 4 weeks (*p* = 0.035 vs. baseline), whereas the placebo drink produced a more modest, non-significant reduction (162 to 141 mg/dL, *p* = 0.078), suggesting that daily kombucha may exert an adjunctive anti-hyperglycemic effect in T2D, albeit in a small, short-duration study. In contrast, evidence for kefir comes mainly from dairy-based products and is more equivocal for glycemic endpoints: In an 8-week parallel RCT in 80 adults with non-alcoholic fatty liver disease, Mohammadi et al. [186] compared a calorie-restricted diet alone vs. the same diet plus 500 mL/day milk kefir. Despite comprehensive assessment of fasting blood sugar, insulin, HOMA-IR, lipids, and inflammatory markers, there were no significant between-group differences in changes in fasting glucose, insulin, or insulin resistance; only HDL-cholesterol and fat-free mass increased significantly in the kefir arm relative to control. A recent meta-analysis of kefir interventions in adults nevertheless reports small but statistically significant pooled reductions in fasting blood glucose (approximately 8 mg/dL) and HOMA-IR, with no consistent effects on anthropometry or lipid profile, underscoring that any glycemic benefit is modest and context-dependent, and that virtually all current evidence derives from dairy kefir rather than plant-based or water kefir formulations.

#### 6.2.2. Soy Ferments (Tempeh/Miso/Natto)

Human data on fermented soy are still limited but converge on two main themes: (i) γ-PGA-rich natto as a viscosity-driven modulator of early postprandial glycemia, and (ii) miso- and tempeh-based low-GI foods that primarily affect lipids and oxidative stress, with modest or neutral effects on conventional glycemic endpoints.

In a pair of randomized crossover meal tests in healthy Japanese adults, white rice alone was compared, rice with low-γ-PGA natto, and rice with high-γ-PGA natto. In the pilot trial, high-γ-PGA natto reduced the 0–120 min glucose iAUC by approximately 20% vs. white rice, with significant attenuation of both glucose and insulin iAUC in the early phase (0–15, 0–30, and 0–45 min), while low-γ-PGA natto produced smaller, mostly non-significant effects [18]. In the subsequent trial focusing on early-phase dynamics, the high-γ-PGA natto again lowered glucose and insulin iAUC vs. low-γ-PGA natto within the first 30–45 min after meal loading, without major differences in later time points, reinforcing a viscosity-mediated slowing of glucose appearance rather than a strong effect on total glycemic exposure [22]. These studies provide proof-of-principle that highly viscous natto can selectively blunt early excursions in postprandial glucose and insulin, a pattern that may be particularly relevant for cardiovascular risk even in otherwise normoglycemic adults.

Building on this mechanistic signal, Papagianni et al. [64] developed a legume-based, carotenoid-enriched miso-type sauce and evaluated it acutely in a crossover RCT in young healthy volunteers (*n* = 14). A high-fat, high-carbohydrate rice meal with the functional miso-type sauce was compared with the same meal containing a non-fermented control sauce. The functional sauce did not significantly modify postprandial glucose, but increased plasma total antioxidant capacity (FRAP) at 3 h, attenuated the rise in triglycerides during the last 1.5 h, and lowered LDL-cholesterol and platelet aggregation vs. baseline, consistent with an oxidative-stress and lipid/platelet-targeted benefit rather than a primary glycemic effect. In a follow-up 30-day randomized crossover pilot in healthy adults (*n* = 10), the same group compared 20 g/day of the bifunctional miso-type sauce with an energy-matched legume-based control; fasting glucose remained unchanged, but the miso-type sauce slightly reduced triglycerides and increased plasma total antioxidant capacity, while both sauces increased LDL-cholesterol, with a smaller increment in the miso arm. Together, these trials suggest that miso-type ferments enriched with carotenoids may contribute more robustly to redox and lipid modulation than to fasting or postprandial glycemia per se [187].

Tempeh-based products appear mostly in the form of low-GI snack prototypes rather than long-term efficacy trials. In a GI study of “tempeh gembus” cookies, Manullang et al. [188] substituted wheat flour with 0, 25, and 50% tempeh gembus flour and measured glycemic responses in vivo alongside in vitro digestibility. Cookies with 50% tempeh flour had the lowest GI (approximately 47, low-GI range) and glycemic load (approximately 6.9 per serving), together with the highest dietary fiber (approximately 25% *w*/*w*) and reduced in vitro starch digestibility, supporting the design of tempeh-rich snacks that minimize postprandial glycemic load while providing ferment-derived fiber and protein. Although this study is small and primarily technological, it illustrates how incorporating tempeh flour into compound foods systematically reduces the GI and GL in humans, in line with in vitro reductions in starch digestibility.

Evidence in patients with T2D is even more limited and often focuses on lipids. In a 4-week RCT in Indonesian adults with T2D (*n* = 64), Fatmah [189] compared two low-GI high-fiber biscuits: a “caromma” biscuit based on modified cassava and koro sword beans, and a “temma” biscuit containing tempeh flour plus date jam. Both biscuits were consumed at 100 g/day. Fasting blood glucose decreased modestly in both groups (approximately 15–25 mg/dL), but changes did not reach statistical significance, whereas total cholesterol, LDL-cholesterol, and triglycerides fell significantly and to a similar extent in both arms, consistent with a high-fiber, low-GI effect in which the specific contribution of tempeh is difficult to isolate.

Finally, large-scale observational data provide complementary, albeit indirect, support for fermented soy within healthy dietary patterns. In a longitudinal cohort of >65,000 Korean adults followed for approximately 5 years, Park et al. [190] showed that an Asian-style balanced diet pattern—characterized in part by higher fermented soy intake and a favorable modified Healthy Eating Index—was associated with greater waist-circumference reduction and lower metabolic syndrome risk, independent of energy intake and polygenic risk scores for abdominal obesity. Although this design cannot attribute effects specifically to fermented soy, it situates natto, miso, and related ferments within a broader metabolic health-promoting dietary context that resonates with the more mechanistic γ-PGA natto and miso-type sauce trials.

#### 6.2.3. Sourdough Breads and Pasta

Human trials with sourdough cereal products are dominated by acute and short-term crossover designs, and collectively point to modest, context-dependent effects on postprandial glycemia. In healthy adults, Chatonidi et al. [191] compared lactic-acid-rich sourdough bread with an otherwise similar yeast-leavened bread and found that sourdough slowed gastric emptying and reduced early postprandial glucose and C-peptide excursions (15–90 min) without altering total glycemic AUC or ad libitum energy intake at a subsequent meal. In a follow-up three-period crossover, the same group tested whole-meal yeast bread, sourdough bread, and a yeast–sourdough bread over three 2-week periods; despite small differences in satiety hormones (PYY, GLP-1, C-peptide), postprandial glucose profiles, gastric emptying, ad libitum energy intake and fecal microbiota composition were essentially comparable across breads [192]. Similarly, Dall’Asta et al. [193] reported no differences in 2 h glucose or insulin iAUC when 12 healthy subjects consumed seven isoglucidic breads differing in wheat genotype (evolutionary vs. modern) and leavening (yeast vs. sourdough). In overweight/obese adults, sourdough pasta did not differ from conventional pasta in acute glycemic or insulinemic responses, nor in OGTT responses after 5-day consumption, although sourdough accelerated gastric emptying and modestly altered fecal SCFA and mycobiome profiles [140]. A recent GI trial in 10 healthy adults showed that manipulating formulation (whole-meal flour, added intact cereals) and baking time of part-baked sourdough breads shifted GI values downward but remained within the medium–high GI range and did not systematically translate into higher satiety iAUC [194]. By contrast, in 12 individuals with T2D monitored with flash glucose sensors, sourdough bread and a functional low-sugar, low-GI bread prepared with “biocrystal” alkaline water produced significantly lower interstitial-glucose AUC_0_–_240_ min, smaller glucose rises at 60–180 min, and lower peak insulin than conventional yeast bread, despite matched carbohydrate loads [36]. Overall, current human data support small sourdough-related improvements in early-phase glycemic and insulinemic responses—particularly when organic acid content is high and/or in T2D—while total glycemic exposure (iAUC), fasting glycemia and longer-term insulin sensitivity remain largely unchanged in short-term trials.

### 6.3. Fermented vs. Non-Fermented Comparators (Head-to-Head)

Only a subset of the studies identified in Section 6.1 and Section 6.2 directly compare a fermented plant matrix with a non-fermented or less-fermented counterpart under controlled conditions. These head-to-head trials are critical to isolate the incremental contribution of fermentation beyond substrate and macronutrient composition and are summarized in Table 3. Overall, they show small but mechanistically informative advantages of selected ferments—especially γ-polyglutamic-acid-rich natto and strongly acidified or low-GI sourdough breads—on early postprandial glucose and insulin dynamics, while many other matrices exert little or no glycemic benefit despite improvements in lipids, oxidative stress, or gut-derived endpoints.

For soy ferments, Araki and colleagues’ natto meal tests exemplify a “clean” comparison: white rice alone vs. rice plus low- vs. high-viscosity natto prepared with different γ-PGA contents. When the carbohydrate load is matched, high-γ-PGA natto consistently blunts early-phase glucose and insulin iAUC relative to both rice alone and low-γ-PGA natto, indicating that fermentation-derived polymers—and the viscosity they confer—can meaningfully reshape early glycemic excursions [18,22]. By contrast, miso-type sauces and tempeh-enriched biscuits are typically compared with non-fermented legume or cereal controls that differ in fiber and protein; in these contexts, acute and 30-day miso-type interventions primarily improve triglycerides, LDL-cholesterol, platelet aggregation, and antioxidant capacity, whereas fasting glucose and postprandial glycemia remain unchanged, and tempeh-based snack prototypes achieve lower GI/GL mainly through higher fiber and altered carbohydrate quality rather than through fermentation per se [64,187,188].

Head-to-head sourdough trials in humans mirror the mixed picture seen in vitro. Acute crossover studies in healthy adults comparing sourdough breads with yeast-leavened breads matched for flour and carbohydrate load show either modest slowing of early postprandial glucose and C-peptide or no discernible difference in total 2 h glucose/insulin iAUC. Short-term (1–2 week) interventions with wholegrain yeast, sourdough, and yeast–sourdough breads similarly report near-identical glycemic profiles, gastric emptying, and ad libitum energy intake, despite clear differences in titratable acidity and organic acid content. Sourdough pasta vs. conventional pasta yields a comparable pattern: minimal acute glycemic differences and no change in OGTT responses after repeated intake, although fecal SCFA and mycobiome features may shift in favor of the sourdough arm. The main exception is a recent crossover study in T2D, where a strongly acidified sourdough bread and a functional low-sugar, low-GI bread both reduced 0–240 min interstitial-glucose AUC and peak insulin compared with a conventional yeast bread, suggesting that in metabolically compromised individuals—and when formulation is explicitly optimized for low GI—the “fermented bread” concept can translate into potentially clinically relevant glycemic improvements, albeit based on small, short-term studies [36,140,193,194].

Among beverages, head-to-head data are even sparser. In adults with T2D, 4 weeks of daily kombucha consumption lowered fasting blood glucose more than a sensory-matched placebo drink, whereas in an 8-week trial in adults with non-alcoholic fatty liver disease, adding dairy kefir to a calorie-restricted diet did not significantly change fasting glucose, insulin, or HOMA-IR compared with diet alone, despite modest benefits on HDL-cholesterol and fat-free mass. Notably, no randomized trials have yet compared plant-based/water kefir with an appropriate non-fermented or differently fermented control on glycemic endpoints [72,186].

Taken together, the head-to-head evidence indicates that fermentation is not a guarantee of glycemic benefit. Meaningful advantages emerge when (i) the fermented and control products are tightly matched for substrate, carbohydrate load, and energy; (ii) fermentation induces large, quantified shifts in viscosity, organic acids, or EPS/β-glucan content; and (iii) participants have impaired glucose regulation (prediabetes or T2D) rather than being young and healthy. These features foreshadow the sources of heterogeneity discussed in Section 6.4 and Section 7 and argue for more deliberately engineered “purpose-built” plant ferments with standardized metabolite and rheology profiles in future trials.

### 6.4. Heterogeneity, Dose–Response, and Safety Signals (Salt, Ethanol, and GI Symptoms)

Across the preclinical and clinical literature, glycemic responses to plant ferments are highly heterogeneous, with few true dose–response studies and substantial variability in matrix, process, and population. Most human trials use single-dose designs (one serving size and one formulation), short durations (acute to 4–12 weeks), and small samples, which limits inferences about “how much” fermented food is needed for a clinically meaningful effect [25,37,195,196]. In practice, the strongest evidence for a graded response comes from γ-PGA-rich natto, where high-γ-PGA natto clearly outperforms low-γ-PGA natto and white rice for early-phase glucose and insulin iAUC in crossover meal tests, despite identical carbohydrate loads. By contrast, miso-type sauces, tempeh-enriched biscuits, sourdough breads/pasta, kombucha, and kefir have been tested at single doses that often differ in fiber, fat or energy from their controls, making it difficult to disentangle fermentation-derived metabolites (organic acids, EPS, peptides) from generic low-GI or high-fiber effects [18,22]. Recent syntheses of fermented fruits/vegetables and kefir trials show small pooled improvements in fasting glucose or HOMA-IR, with wide confidence intervals and high between-study heterogeneity, underscoring that effects are modest and context-dependent rather than uniform [37,88,195].

#### 6.4.1. Salt Load from Fermented Vegetables

Kimchi and related salt-fermented vegetables raise obvious concerns about sodium exposure, particularly when consumed daily in 100–200 g portions. However, epidemiologic and intervention data suggest a more nuanced picture. Cross-sectional and 12-year cohort analyses in Korean adults report that high kimchi intake (≥145–200 g/day) is not associated with higher hypertension prevalence or incident risk in the general population, with a possible exception of watery kimchi in obese men; authors hypothesize that high potassium, fiber, and vegetable content may partially offset sodium’s pressor effect [197,198]. In spontaneously hypertensive rats, high-sodium kimchi further elevated BP, whereas low-sodium kimchi did not differ from control diet, supporting reformulation as a viable strategy [199]. A recent scoping review of kimchi trials concludes that short-term human interventions (typically 50–200 g/day for up to 12 weeks) do not worsen BP and, in some cases, modestly lower systolic/diastolic values when kimchi replaces other salted foods. For metabolic trials, this means that sodium content and potassium balance should be explicitly reported and, where possible, low-sodium and/or potassium-enriched formulations should be prioritized in individuals with hypertension or high cardiometabolic risk [56,58].

#### 6.4.2. Ethanol Exposure from Kombucha

Ethanol is an underappreciated safety and regulatory issue for kombucha and other sugar-fermented beverages. Under controlled conditions, commercial kombucha typically contains <0.5% alcohol by volume (ABV), but surveys of retail products and home brews have repeatedly documented batches exceeding 0.5–1.0% ABV and, in some cases, reaching approximately 2–3% ABV over the shelf life due to continued fermentation [200]. Regulatory guidance in several jurisdictions now requires producers to monitor ABV over the stated shelf life, keep values below 0.5–1.0% depending on alcohol legislation, and clearly label alcohol content and refrigeration instructions [201]. Case reports and toxicology reviews describe rare but serious adverse events with improperly brewed, unregulated kombucha (lactic acidosis, hepatotoxicity, heavy-metal exposure), typically linked to home production rather than commercial beverages. The randomized pilot in T2D reporting improved fasting glycemia noted no serious adverse events over 4 weeks, but its small size and short duration preclude firm safety conclusions [71]. Future kombucha and similar beverage trials should routinely quantify ethanol and organic acids, report ABV in publications, and define exclusion criteria for children, pregnancy, and individuals with alcohol use disorders.

#### 6.4.3. Gastrointestinal Symptoms and Tolerability

Overall, lactic-fermented plant foods show a favorable GI safety profile, with most RCTs reporting no serious adverse events and only mild, transient symptoms (bloating, gas, changes in stool frequency) when fermented products are introduced [24]. In the natto, miso-type sauce, sourdough, and kombucha trials discussed above, dropout due to GI intolerance was rare, and adverse events were comparable between fermented and control arms [22,64,140,193,202]. A recent systematic review of fermented-food interventions found that, across diverse matrices, fermented foods tend to improve bowel-movement frequency, stool consistency, and functional GI symptoms, with occasional increases in flatulence during the first weeks of consumption [24]. Nevertheless, gas-producing substrates (cabbage, wheat) combined with microbial fermentation can exacerbate bloating or pain in individuals with irritable bowel syndrome or small intestinal bacterial overgrowth; for such patients, slow titration of dose and careful matrix choice may be warranted [203]. From a reporting perspective, standardizing GI adverse-event capture (e.g., Rome IV-aligned symptom diaries) and including tolerability as a predefined secondary endpoint would materially strengthen the safety evidence base [204].

In summary, heterogeneity in dose, duration, matrix composition, and safety profiles complicates interpretation of glycemic signals from plant ferments. Where benefits are seen, they tend to arise from well-characterized, “high-intensity” ferments (e.g., high-γ-PGA natto, strongly acidified or low-GI sourdough, carefully brewed kombucha) and in participants with impaired glucose regulation. At the same time, sodium load from fermented vegetables and ethanol from kombucha remain important safety considerations, especially for vulnerable groups. These themes foreshadow the methodological and reporting gaps addressed in Section 7 and point toward the need for multi-arm, dose-ranging trials with rigorous metabolite and safety characterization.

## 7. Sources of Heterogeneity and Methodological Gaps

### 7.1. Starter/Strain Variability and Process Parameters

Across plant ferments, the same nominal matrix (“kimchi”, “miso”, “sourdough”, “kombucha”) can conceal wide variation in microbial starters, fermentation time–temperature profiles, and back-slopping practices. Spontaneous or household ferments often rely on undefined consortia whose taxonomic and functional composition can shift over successive batches, while commercial products increasingly use proprietary mixed starters that are rarely disclosed in detail [205]. Even when putative “beneficial” taxa (e.g., *L. plantarum*, *L. mesenteroides*, *B. subtilis*) are shared, strain-level differences in EPS production, acidification rate, proteolytic activity, or phytate degradation can markedly alter viscosity, organic acid profiles, peptide release, and ultimately glycemic effects [206]. Process parameters such as dough yield, hydration, fermentation duration, and proofing/baking conditions for sourdough; salt concentration and temperature for kimchi; and sugar concentration, tea type, and fermentation/conditioning time for kombucha further modulate metabolite output [207]. Yet most intervention trials describe the ferment only generically and rarely standardize or validate starter performance across production runs, making it difficult to compare or reproduce findings. This under-reporting is especially problematic for polymer-linked mechanisms summarized in Section 4.2.4 and Table 1, where small changes in strain or process can substantially change EPS, BC, or γ-PGA yield and rheology.

### 7.2. Substrate Composition and Co-Ingredients (Polyphenols/Fiber/Salt)

Matrix effects are equally important. Fermented vegetables, soy pastes, kombucha, kefir, and sourdough are built on substrates that differ substantially in intrinsic fiber, RS, protein, lipid, mineral content, and polyphenolic profile. In many trials, the fermented product is compared with a non-fermented control that is not fully matched for these attributes—tempeh-enriched biscuits introduce more protein and fiber than control biscuits; sourdough breads differ in wholegrain content or added seeds; miso-type sauces add carotenoid-rich fruit extracts; kombucha adds organic acids and small amounts of residual sugars compared with water or soft drinks [196]. This blurs mechanistic attribution: Improved glycemia, lipids, or satiety may stem as much from altered carbohydrate quality, fiber content, or co-delivered bioactives as from fermentation-derived metabolites per se.

Co-ingredients introduce additional layers of heterogeneity. Polyphenol-rich additions (fruit peels, teas, herbs, spices) can modulate α-amylase/α-glucosidase inhibition and gut microbial metabolism; high sodium in kimchi and pickled vegetables interacts with potassium and nitrate content of the vegetable base; added fats and proteins influence gastric emptying and incretin responses [205]. Very few studies intentionally factorialize these elements (e.g., fermented vs. non-fermented × high vs. low fiber, or fermented matrix with vs. without phenolic enrichment), and most do not report full nutrient and non-nutrient composition alongside fermentation parameters. As a result, teasing apart “fermentation effects” from substrate and co-ingredient effects remains a major methodological gap [35].

### 7.3. Analytical Standardization (Minimum Panel, Multi-Omics, SCFA Tracers)

The biochemical characterization of plant ferments and their in vivo footprints is strikingly non-standardized. Many interventions report only pH or titratable acidity; fewer quantify lactic, acetic, and other organic acids, residual sugars, ethanol, salt, or γ-PGA/EPS content; and only a minority provide detailed phenolic or peptide profiles [35]. On the host side, plasma SCFA, bile acids, gut hormones, and inflammatory markers are inconsistently measured, and microbiome analyses often stop at 16S-level taxonomic shifts without integrating metagenomics, metabolomics, or flux information [10]. This hampers mechanistic interpretation and comparison across studies.

A pragmatic way forward is to agree on a minimum analytical panel for fermented test products (at least pH, titratable acidity, major organic acids, residual sugars, sodium/potassium, rough EPS/γ-PGA, or β-glucan content) plus a small set of host readouts (fasting and postprandial glucose/insulin, a standard lipid panel, at least one SCFA or bile-acid proxy, and gut-hormone or inflammatory markers where feasible) [37]. Table 1 and Table 2 in this review outline such minimum reporting sets for polymer-linked mechanisms and glycemic endpoints. For mechanistic studies, integrating multi-omics (microbiome–metabolome–host) and using stable-isotope tracers for SCFA or glucose flux would substantially strengthen causal inferences but is rarely implemented at present. As noted in Section 2.10, targeted DOI sets for rheology and analytical anchors can complement intervention data by bridging matrix properties to enzyme-level and clinical outcomes, provided that results are reported in a standardized, comparable format.

### 7.4. Clinical Design: Background Diet, Duration, Statistical Power, Standardized Endpoints

Methodological limitations at the trial level further amplify heterogeneity. Most studies are acute or short-term (hours to 4–12 weeks), include small samples (often *n* < 30 per arm), and are underpowered to detect modest but clinically relevant changes in glycemia or insulin sensitivity, particularly when multiple secondary endpoints are measured without adjustment [35]. Background diet is typically only lightly controlled or monitored, meaning that participants may vary widely in habitual fiber, wholegrain, sodium, and alcohol intake—all of which can modify the effect of fermented foods. Few trials stratify or adjust for baseline glycemic status, microbiome composition, or medication use, even though signals tend to be stronger in individuals with prediabetes or T2D than in healthy young adults [37].

Endpoints and outcome definitions are also heterogeneous. Some studies focus on fasting glucose or HOMA-IR, others on OGTT/iAUC, GI, CGM-derived metrics, or composite cardiometabolic scores; sampling schedules and analytical methods differ, making meta-analysis challenging [197]. Gastrointestinal symptoms and tolerability, as discussed in Section 6.4, are rarely prespecified and often captured only informally, and safety characterization (e.g., sodium burden, ethanol content) is inconsistent [25]. Going forward, trials of plant ferments for metabolic health would benefit from (i) adequately powered, parallel, or well-designed crossover studies with clearly defined primary endpoints; (ii) standardized core outcome sets for glycemic control (e.g., fasting glucose, HbA1c where duration allows, OGTT, or CGM-based metrics) and safety/tolerability; (iii) tighter control or at least rigorous monitoring of background diet; and (iv) prespecified subgroup analyses by metabolic status and, where possible, microbiome-defined phenotypes [24]. These design improvements—together with the analytical minimum sets proposed in Table 1 and Table 2—are essential to move from intriguing but heterogeneous signals to robust, generalizable evidence on fermented plant foods and glycemic control.

Taken together, these sources of heterogeneity make it clear that “fermented” does not automatically equate to “metabolically superior.” Glycemic benefits emerge most consistently when (i) the fermented product is purpose-built and well-characterized (matrix, process, polymers, and acids, key phytochemicals), (ii) the comparator is tightly matched for substrate, carbohydrate load, and energy, and (iii) participants have impaired or at-risk glycemic profiles rather than being young, healthy volunteers. Conversely, loosely defined ferments, poorly matched controls, and underpowered, short-duration trials tend to yield neutral or inconsistent results. These insights underpin the study-design recommendations in Section 8, where we outline practical steps for engineering and testing plant-based fermented foods in a way that maximizes mechanistic interpretability and clinical relevance for glycemic control.

## 8. Recommendations for Future Research

### 8.1. Minimum Reporting Standards for Fermented Foods and Postbiotics in Trials

Across the current literature, many interventions describe the test product only generically as “kimchi”, “miso”, “sourdough bread”, or “kombucha”, with minimal information on starters, process parameters, or metabolite profiles, despite repeated calls for clearer definitions and characterization of fermented foods and postbiotics [8,10,35,37]. The ISAPP postbiotic consensus further emphasizes that products should be explicitly classified as live fermented foods vs. postbiotic preparations, with their composition and inactivation processes clearly described [9].

Future trials should therefore adopt minimum reporting standards that include (i) detailed substrate and formulation (cereal/vegetable/soy base, whole vs. refined, added fiber, fat, polyphenol-rich ingredients); (ii) starter information (at least genus/species, with strain identifiers and deposition where feasible) and core process parameters (salt concentration, fermentation time and temperature, dough yield/hydration, proofing/baking conditions, storage); and (iii) a core analytical panel for the fermented product comprising pH, titratable acidity, major organic acids, residual sugars and ethanol, sodium and potassium, and—where relevant—approximate concentrations of γ-PGA, EPS/β-glucans, or other targeted postbiotic structures [8,10,35].

These product-level descriptors should be aligned with the polymer-focused metrics (Table 1) and the analytical anchors for enzyme-to-metabolite routes (Table 2), so that mechanistic and clinical data can be interpreted on the same grid.

On the host side, alignment with the ISAPP framework implies that studies should state whether they are testing a fermented food (live microbes plus metabolites) or a postbiotic (inactivated microbes/components) and prespecify primary outcomes that demonstrate health benefit in the relevant population [9]. For metabolic trials, a pragmatic “minimum set” would include fasting glucose and insulin (with HOMA-type indices), at least one standardized postprandial metric (OGTT, mixed-meal test, or CGM-based AUC/peak), and routine safety markers such as lipids and BP [10,37]. By analogy with CONSORT extensions for probiotics and other nutrition interventions, such standards would immediately improve comparability and meta-analytic synthesis.

### 8.2. Integrated Metabolomics–Microbiome–Host-Omics in Interventions

Most fermented-food trials still report clinical endpoints with sparse mechanistic data. Reviews of fermented foods and cardiometabolic health highlight that, although changes in microbiota composition are frequently described, integration with metabolomics and host biology remains rare [8,11,35]. At the same time, multi-omics intervention studies in other dietary contexts—such as high-fiber or Mediterranean-style diets—are beginning to show how diet reshapes microbial functions, circulating metabolites (SCFAs, bile acids, aromatic metabolites), and organ-level metabolism in concert [1,28].

For plant ferments and postbiotics, future RCTs should, where feasible, embed (i) high-resolution microbiome profiling (shotgun metagenomics or at least 16S/ITS with functional inference) to capture taxa and pathways responsive to specific matrices; (ii) targeted and untargeted metabolomics of SCFAs, bile acids, amino-acid derivatives, and phenolic conjugates in plasma/urine; and (iii) limited host-omics such as transcriptomics or phospho-proteomics in PBMCs in mechanistic subcohorts [1,11].

Conceptual work on microbiome–host metabolic crosstalk and flux further suggests that stable-isotope tracers (for SCFA or glucose flux) could be layered onto these designs to move from correlation to causal nutrient–microbe–host pathways [2]. Even if full multi-omics cannot be implemented in all participants, nested substudies within larger glycemic RCTs—using the analytical anchors summarized in Table 2—would substantially strengthen mechanistic interpretation.

### 8.3. Trial Designs: Factorial and Adaptive Approaches, Microbiome/Metabolic Stratification

Methodologically, most fermented-food studies still use simple acute or short-term crossover designs with one dose of one product vs. one control, modest sample sizes, and heterogeneous endpoints [35,37]. Lessons from precision-nutrition and glycemic-response research indicate that more sophisticated designs are both feasible and informative. Large CGM-based cohorts have demonstrated high interpersonal variability in postprandial glucose responses and the ability of machine-learning models that integrate diet, clinical parameters, and microbiome features to predict these responses and guide individualized diets [3,4].

For plant ferments, factorial designs offer a powerful way to disentangle fermentation from matrix and co-ingredients—for example, randomizing participants to fermented vs. non-fermented versions crossed with high vs. low fiber, or with vs. without added polyphenol extracts. Adaptive and response-enriched designs could use early CGM or metabolomic readouts to preferentially enroll “responders” in later trial phases, mirroring adaptive strategies already applied in precision-glycemia studies [3].

Given that responses to diet are shaped by baseline metabolic status and microbiome composition, future trials should predefine stratification or subgroup analyses by glycemic status (normoglycemia, prediabetes, T2D) and, where possible, microbiome strata or functional clusters [1]. Incorporating CGM-derived endpoints (e.g., 24-h AUC, time-above-range, early postprandial increments) alongside fasting measures in at least a subset of participants would capture subtler effects of plant ferments on glycemic variability and early-phase excursions than fasting glucose or HOMA-IR alone.

### 8.4. Path-to-Product: Toward Purpose-Built Plant Ferments for Metabolic Health

Finally, the field needs to move from studying heterogeneous traditional ferments “as found” to developing purpose-built plant ferments and postbiotic preparations with predefined metabolic targets. Conceptual and experimental work now frames fermented foods as functional microbial systems whose composition and performance can be engineered via starter selection, process control, and data-driven design [8,11]. Recent tools such as the Microbial Food DataBase (MiFoDB) and metagenomics-based workflows for fermented-food profiling enable strain-level mapping of microbial consortia and associated metabolite outputs, providing a technical basis for rational starter and substrate design [10].

For metabolic health, a plausible pipeline would involve (i) mechanistic screens in vitro and in animal models to identify polymer (γ-PGA, EPS/β-glucan), peptide, or organic-acid signatures linked to improved glycemic and insulin dynamics; (ii) translation of these signatures into scalable fermentation processes with tight control of strains, substrate, salt, and sugar levels and verification that postbiotic profiles (e.g., viscosity, acid, and ethanol content) are reproducible and compatible with sodium and alcohol guidelines; and (iii) iterative human studies, starting with acute CGM studies and progressing to longer RCTs in prediabetes and T2D, using well-matched non-fermented comparators and the reporting/design standards outlined above [11,37].

The ISAPP postbiotic definition provides a conceptual and regulatory anchor for such preparations—as “preparations of inanimate microorganisms and/or their components that confer a health benefit on the host” [9]—and is particularly relevant for shelf-stable, low-sodium, low-ethanol products intended for glycemic control. By integrating microbial ecology, fermentation technology, multi-omics, and rigorous clinical trial methodology, future work can move toward a new generation of engineered plant ferments that deliver quantifiable, reproducible benefits for glycemic control and broader cardiometabolic health [1,8,11].

## 9. Conclusions

### 9.1. Synthesis of Evidence and Translational Implications

Across mechanistic, preclinical, and clinical layers, plant-based fermented foods emerge less as a single “category” and more as a spectrum of matrices and postbiotic profiles with highly context-dependent effects on glycemic control. In vitro and animal data consistently show that lactic and acetic fermentation of vegetables, soy, and cereals can (i) remodel carbohydrate structure (more SDS and RS, less RDS); (ii) enrich matrices with organic acids, EPS/γ-PGA, β-glucans, peptides, and remodeled phenolics; and (iii) improve barrier integrity, inflammatory tone, bile acid, and SCFA signaling. These mechanisms plausibly support better glucose handling and insulin sensitivity, especially under obesogenic or inflammatory conditions.

Human intervention data, by contrast, are heterogeneous and generally modest in magnitude. The clearest glycemic signal comes from γ-PGA-rich natto, where high-viscosity natto reproducibly blunts early postprandial glucose and insulin excursions compared with low-γ-PGA natto or non-fermented rice, despite identical carbohydrate loads. Sourdough breads and pasta, miso-type sauces, tempeh-enriched snacks, and kombucha show more variable outcomes: small improvements in early-phase glycemia or insulin, or in lipids, oxidative stress, and liver markers, often without large changes in total glycemic exposure (iAUC) or fasting indices. In T2D and prediabetes, the likelihood of seeing a benefit appears higher, particularly when products are explicitly designed for low GI and quantified postbiotic content. Overall, the translational message is that fermentation can tilt carbohydrate quality and postprandial dynamics in a favorable direction, but effects are not guaranteed and depend on matrix, process, and host phenotype.

### 9.2. Clinical Nutrition and Nutraceutical Development

From a clinical nutrition perspective, plant ferments fit most naturally as adjuncts to established cardiometabolic dietary patterns rather than as stand-alone therapies. When incorporated into vegetable-rich, high-fiber, minimally processed diets, kimchi and other fermented vegetables, natto/miso, and wholegrain sourdoughs can contribute additional fiber, potassium, bioactive peptides, and microbial metabolites, potentially improving satiety, lipid profiles, and early postprandial responses without worsening overall sodium or glycemic load—provided that salt and portion size are managed and ethanol remains low. Kombucha and kefir-like beverages may offer incremental benefits in selected patients but require careful attention to alcohol content, added sugars, and GI tolerability.

For product development, the evidence supports a pivot away from vaguely defined “traditional” ferments toward standardized, purpose-built fermented foods and postbiotic preparations. This means specifying strains and process conditions; quantifying key metabolites (acids, EPS/γ-PGA, ethanol, sodium, phenolics, peptides); and designing formulations that deliver clinically meaningful changes in viscosity, GI, or postprandial excursions while meeting safety constraints (salt and alcohol). Postbiotic concepts—heat-treated ferments, cell-free supernatants, or purified polymers—open additional routes for shelf-stable, low-sodium, low-ethanol products that can be tested like nutraceuticals, with clear dosing, quality control, and regulatory positioning. However, given the modest effect sizes observed to date, such products are likely to be adjunctive tools embedded within broader lifestyle and pharmacologic strategies for metabolic disease rather than primary therapies.

### 9.3. Personalization Opportunities (Microbiome and Metabolic Phenotype)

Finally, the emerging interface between plant ferments, the gut microbiome, and metabolic phenotype suggests real, but still largely unrealized, opportunities for personalization. Interindividual variability in glycemic responses to standard foods is now well documented, and baseline microbiome composition, habitual diet, and insulin sensitivity all plausibly modulate response to fermented matrices and postbiotics. Signals from existing trials already hint that benefits are stronger in individuals with impaired glucose regulation than in healthy young adults, and that shifts in taxa such as *Akkermansia*, *Bifidobacterium*, and butyrate producers may track with improved metabolic readouts.

Translating these observations into practice will require trials that deliberately stratify by metabolic status, incorporate CGM and microbiome profiling, and test whether specific fermented foods or postbiotic signatures can be matched to “responder” phenotypes. In the longer term, one could envision precision-nutrition frameworks in which engineered plant ferments—tailored for viscosity, SCFA, or bile-acid modulation, or peptide release—are selected based on an individual’s microbiome–metabolic profile and integrated into personalized dietary prescriptions. At present, however, the evidence base supports a more cautious conclusion: Plant-based fermented foods are promising tools to fine-tune glycemic and cardiometabolic risk within healthy dietary patterns, but robust, stratified clinical trials are still needed before personalized, fermentation-centric prescriptions can be routinely recommended.

## Figures and Tables

**Table 1:** Minimum reporting set for polymer-linked glycemic mechanisms in fermented plant foods (EPS, BC, and γ-PGA).

Item	What to Report (at Minimum)	Why it Matters (Mechanistic Link)	Example Metrics and Sources
Polymer dose and identity in the edible matrix	EPS (g/L or % *w*/*w*) and type (dextran/levan/HePS); BC pellicle mass/thickness or dispersed-fiber content; γ-PGA (g/serving)	Polymer mass sets baseline viscosity and network formation, controlling diffusion and enzyme access; for γ-PGA, dose relates to the magnitude of early glycemic attenuation seen in natto meal tests	EPS yield vs. temperature/medium [38,39]; KBC content effects [40]; γ-PGA titre/dose context and natto trials [41,42,43].
Polymer size (or proxy)	MW distribution (e.g., SEC-MALS), intrinsic viscosity, or fractionation; for modified celluloses, degree of substitution	Larger/entangled chains and substituted networks increase shear-thinning and elasticity → stronger mass-transfer resistance	Levan size → rheology [31]. Modified BC (carboxymethyl-BC) structure–flow relationships [44].
Rheology at digestion-relevant conditions	Flow curves (apparent viscosity vs. shear rate approximately 1–1000/s) and, when feasible, G′/G″ at 25–37 °C; note temperature dependence	Captures diffusion control under gastric/intestinal shear rates; temperature shifts viscosity	Levan flow behavior [31]; KBC–chitosan shear-thinning and temperature dependence [40].
Fermentation/process parameters	Substrate (e.g., sucrose, flour type), pH/temperature profile, oxygen transfer/aeration, salts/ions; time course	Process conditions tune polymer yield, MW, and conformation → changes in matrix viscosity and diffusion	EPS vs. temperature/medium [38,39]; γ-PGA bioprocess levers [41,42,43].
Matrix co-modulators	Ethanol and organic acids (kombucha), salts/ionic strength, added hydrocolloids, proteins	Co-polymers and ionic milieu shift polymer conformation and enzyme access; protein–polysaccharide interactions can amplify viscosity	Protein–polysaccharide thickening and microstructure changes [45]; KBC blends biopolymers [40].
Mechanistic digestion readouts	α-Amylase/α-glucosidase activity and/or starch hydrolysis curves measured on the actual polymer-containing matrix	Links polymer-driven rheology to enzyme kinetics in situ, rather than inferring from viscosity alone	Study-design guidance in EPS/kefir and sourdough contexts [38].
Postprandial endpoints	iAUC_0–120_ glucose (± insulin), sampling schedule, meal composition and matched CHO load, polymer dose per serving	Detects early-phase glycemic dampening expected from viscosity/diffusion limits; allows comparison with γ-PGA natto “positive control” data	Reporting framework aligned with polymer metrics (methods rationale) [38].
Transparent data alignment	A compact summary aligning polymer dose → viscosity/G′/G″ → enzyme readout → glycemic outcome; include raw rheograms where feasible	Enables cross-study comparisons and supports causal attribution from polymer physics to glycemic response	Examples of size → rheology (levan) and loading → viscosity (KBC blends) [31,40], plus EPS process effects [38].

Abbreviations: EPS, exopolysaccharide; KBC, kombucha bacterial cellulose; SEC-MALS, Size-Exclusion Chromatography with Multi-Angle Light Scattering; BC, bacterial cellulose; MW, molecular weight; γ-PGA, Poly-γ-glutamic acid; iAUC_0–120_, incremental area under the curve.

**Table 2:** Analytical anchors and minimal reporting set to align enzyme-level mechanisms with metabolite dynamics and clinical endpoints in fermented plant food studies.

Pathway/Topic	Primary Targets	Matrix and Time Window	Primary Method (One-Liner)
Organic acids and residual sugars and ethanol	Acetate, lactate, citrate/malate; glucose, fructose, sucrose; ethanol	Food/beverage (batch and serving)	HPLC-UV/RI (matrix-matched calibration); report pH and titratable acidity † [46].
SCFAs	Acetate, propionate, butyrate	Plasma 0–120 min; urine 0–240 min	GC–MS (propyl-chloroformate) or LC–MS/MS (3-NPH); scheduled MRM ‡ [47].
Phenolics and low-MW catabolites	Phenolic acids; urolithins/PGVL; indole-3-lactic/propionic	Food + plasma/urine 0–120 (240) min	LC–MS/MS (MRM); with/without enzymatic deconjugation; control matrix effects † [48].
Isoflavones (if soy/plant milks)	Genistein, daidzein (free and conjugates)	Product ± plasma 0–120 min (if targeted)	UPLC–MS/MS (often negative mode); report MRM transitions; SIL-IS ‡ [48].
Process covariates (transport surrogates)	EPS (g/L), viscosity/flow curves; BC mass/thickness	Food/beverage at serving ‖	Phenol–sulfuric (EPS proxy); rheometry; gravimetry/thickness (BC) [45,49,50].

† Minimal validation to report: linearity (r^2^), LOD/LOQ, precision, recovery, matrix effect, stability. ‡ Internal standards: ^13C^-acetate/propionate/butyrate for SCFAs; SIL-IS for isoflavones (e.g., ^13C^/^2H^-genistein/daidzein). ‖ Record fermentation conditions (substrate, pH, temperature); use these as covariates in the models (e.g., regression/SEM). Abbreviations: HPLC-UV/RI, High-Performance Liquid Chromatography with Ultraviolet (UV) and Refractive Index (RI) detectors; SCFA, short-chain fatty acids; GC–MS, Gas Chromatography–Mass Spectrometry; LC–MS/MS, Liquid Chromatography–Tandem Mass Spectrometry; 3-NPH, 3-nitrophenylhydrazine; MRM, multiple reaction monitoring; MW, molecular weight; PGVL, phenyl-γ-valerolactones; UPLC, Ultra-Performance Liquid Chromatography; SIL-IS, stable-isotope-labeled internal standards; EPS, exopolysaccharides; BC, bacterial cellulose.

**Table 3:** Fermented vs. non-fermented plant matrices: human trials with glycemic endpoints.

Matrix/Comparator	Population and Design	Fermentation/Metabolite Characterization	Glycemic Endpoints	Main Result vs. Non-Fermented/Less-Fermented Control
High- vs. low-γ-PGA natto + white rice vs. white rice alone	Healthy Japanese adults; two acute randomized crossover meal tests [18,22]	Natto prepared with different *B. subtilis* strains to yield low- vs. high γ-PGA; viscosity and γ-PGA concentration quantified; rice portion standardized.	0–120 min plasma glucose and insulin iAUC; early-phase (0–15/30/45 min) iAUC.	High-γ-PGA natto blunted early (0–30/45 min) glucose and insulin iAUC vs. both white rice alone and low-γ-PGA natto; total 0–120 min glucose iAUC modestly reduced. Effects attributed to fermentation-derived γ-PGA and increased meal viscosity.
Miso-type fermented sauce vs. non-fermented legume-based control sauce (acute)	Healthy young adults (*n* = 14); single-meal randomized, single-blind crossover trial [64]	Miso-type sauce produced by *Aspergillus oryzae* fermentation and enriched with carotenoid-rich fruit by-products; higher total phenolics, carotenoids, and antioxidant capacity than control; meals otherwise isocaloric and macronutrient-matched.	Postprandial serum glucose (0–4 h); secondary: triglycerides, LDL-cholesterol, total antioxidant capacity, platelet aggregation.	No significant difference in postprandial glucose or glucose iAUC; functional miso sauce increased plasma antioxidant capacity, attenuated late-phase triglyceride rise, lowered LDL-cholesterol acutely, and reduced platelet aggregation vs. control.
Miso-type fermented sauce vs. non-fermented control (30-day intake)	Healthy adults (*n* = 10); 2 × 30-day crossover RCT with 20 g/day of miso-type vs. control sauce [187]	Same miso-type and legume-based control sauces as above; carotenoid- and phenolic-enriched fermented matrix vs. non-fermented.	Fasting glucose (primary glycemic marker); secondary: lipids, antioxidant status.	Fasting glucose remained unchanged in both periods; miso-type sauce modestly reduced triglycerides and increased plasma antioxidant capacity relative to control.
Tempeh-enriched biscuits/cookies vs. non-tempeh controls	Healthy adults; acute GI testing of snack products [188,189]	Partial replacement of wheat or cassava flours with tempeh (fermented soybean) flour; higher protein and dietary fiber in tempeh formulations; no explicit EPS/organic-acid quantification.	Capillary glucose curves over 2 h; calculated GI and GL.	Tempeh-rich snacks showed lower GI and GL than non-tempeh controls, largely attributable to higher fiber and altered starch/protein composition; fermentation-derived components were not isolated mechanistically.
Sourdough vs. yeast-leavened wheat bread (acute)	Healthy adults; single-meal crossover RCT [191]	Same wheat flour and recipe; sourdough bread produced with LAB showing higher titratable acidity and lactic/acetic acid content and lower pH than yeast bread.	Postprandial plasma glucose, C-peptide, and insulin (0–180 min); gastric emptying; satiety ratings.	Sourdough bread slightly delayed gastric emptying and attenuated early (15–90 min) glucose and C-peptide responses vs. yeast bread, but total 2 h glucose and insulin iAUC were not significantly different.
Wholegrain yeast bread, sourdough bread and yeast–sourdough bread	Healthy adults; three-period 2-week crossover (each bread for 14 days) with standardized test meals [192]	All breads based on whole-meal flour; leavening varied (yeast, sourdough, yeast–sourdough); sourdough breads had higher titratable acidity and organic acid content.	Standardized meal glucose and insulin responses; gastric emptying; and libitum energy intake; satiety hormones.	Glycemic profiles, gastric emptying and energy intake were essentially similar across breads; only small and inconsistent differences in satiety hormones (GLP-1, PYY, C-peptide).
Sourdough breads and pasta vs. conventional yeast-leavened counterparts	Healthy adults in acute GI/OGTT experiments and short-term pasta interventions:	Sourdough products characterized by higher acidity and, in some cases, modestly higher resistant/SDS; controls were yeast-leavened or non-fermented, matched for available carbohydrate.	2 h glucose and insulin iAUC; GI; OGTT after repeated intake.	Across multiple cereal products, sourdough rarely produced substantial differences in overall postprandial glycemia or insulinemia vs. conventional products; GI often shifted from high to medium–high but without clear translation into lower iAUC.
	– Seven isoglucidic breads differing in wheat genotype and leavening [193] – Sourdough vs. conventional pasta, acute + 5-day consumption [140]. – Recent GI testing of part-baked sourdough breads with varied formulation [194]			
Sourdough bread and functional low-GI bread vs. conventional yeast bread	Adults with T2D; acute randomized crossover study with continuous glucose monitoring [36]	Sourdough bread with higher acidity and organic acids; functional bread additionally formulated with reduced available sugars and higher fiber/“biocrystal” water; all breads matched for total carbohydrate load.	Interstitial glucose profiles (0–240 min; CGM); peak glucose; insulin responses.	Both sourdough and functional low-GI bread reduced 0–240 min glucose AUC, postprandial glucose rise, and peak insulin vs. conventional yeast bread, indicating potentially clinically relevant benefits when formulation is optimized and participants have T2D, although evidence is still limited to small, short-term trials.
Kombucha vs. placebo beverage	Adults with T2D (*n* = 12); double-blind crossover RCT, 4 weeks kombucha vs. 4 weeks placebo with washout [72]	Unpasteurized kombucha produced by SCOBY fermentation of tea; organic acids, pH, and residual sugars characterized; placebo beverage matched for flavor and appearance but unfermented.	Fasting blood glucose (primary); HbA1c and other metabolic markers exploratory.	Kombucha reduced fasting blood glucose significantly vs. baseline (from approximately 164 to 116 mg/dL), whereas the placebo drink produced a smaller, non-significant reduction; between-treatment comparison favored kombucha in this small pilot, but postprandial responses were not systematically assessed.
Milk kefir plus calorie-restricted diet vs. calorie-restricted diet alone	Adults with non-alcoholic fatty liver disease (*n* = 80); 8-week parallel-group RCT [186]	Daily 500 mL dairy kefir added to hypocaloric diet; kefir characterized by pH, titratable acidity, and viable counts of LAB and yeasts; control group received diet only.	Fasting glucose, fasting insulin, HOMA-IR; secondary: lipids, anthropometry, inflammatory markers.	No significant between-group differences in changes in fasting glucose, insulin, or HOMA-IR; kefir group showed improvements in HDL-cholesterol and fat-free mass vs. diet alone, suggesting limited additional glycemic benefit in this context.

Abbreviations: γ-PGA, Poly-γ-glutamic acid; iAUC, incremental area under the curve; LDL, low-density lipoprotein; RCT, randomized controlled trial; GI, glycemic Index; EPS, exopolysaccharides; GL, glycemic load; LAB, lactic acid bacteria; GLP-1, Glucagon-like Peptide-1; PYY, Peptide YY; OGTT, Oral Glucose Tolerance Test; SDS, slowly digestible starch; T2D, type 2 diabetes; CGM, Continuous Glucose Monitoring,; SCOBY, Symbiotic Culture of Bacteria and Yeast; HbA1c, hemoglobin A1c; HOMA-IR, Homeostasis Model Assessment for Insulin Resistance; HDL, high-density lipoprotein.

## Data Availability

No new data were created or analyzed in this study. Data sharing is not applicable to this article.

## Supplementary Materials

The following supporting information can be downloaded at: https://www.mdpi.com/article/10.3390/molecules31020360/s1, Table S1: Plant-based fermented foods relevant to glycemic control.
