# Supplementary material for: Fermented Plant-Based Foods and Postbiotics for Glycemic Control—Microbial Biotransformation of Phytochemicals
Source: Molecules. 2026 Jan 20;31(2):360. doi: 10.3390/molecules31020360 (PMC12844311; doi:10.3390/molecules31020360)
Supplement: Supplementary file 1 [file molecules-31-00360-s001.zip › molecules-4021940-supplementary.pdf]

Table S1. Plant-based fermented foods relevant to glycemic control.

Fermented food (plant-based)	Primary substrate/matrix	Predominant microbes (typical)	Key biotransformed phytochemicals	Characteristic postbiotics/metabolites	Standardization fields
Kimchi (fermented *Brassica* vegetables)	Chinese/Napa cabbage; radish; spices; garlic/ginger; chili	*Leuconostoc spp*. (early); *Lactiplantibacillus/Lactobacillus spp*. (later); *Weissella spp.*	Phenolic acids; peptides; (context-dependent) glucosinolate derivatives; GABA (in GABA-enriched processes)	Mannitol; lactic and acetic acids; EPS; peptides	pH; titratable acidity (g/L lactic acid); salt %; fermentation time/temperature; predominant taxa (16S/shotgun); residual sugars; organic acid profile (HPLC); mannitol; EPS (g/L); viable LAB/yeast counts (CFU/g); recipe (vegetable:spice ratios) (12, 13, 17, 21, 38-43)
Other fermented vegetables (e.g., sauerkraut, pickled vegetables)	Cabbage and assorted vegetables; brine	*Leuconostoc spp*.; *Lactobacillus/Lactiplantibacillus spp*.; *Pediococcus spp.*	Enhanced phenolic acid availability; partial deconjugation of flavonoids	Lactic and acetic acids; mannitol; CO_2; EPS (strain-dependent)	pH; titratable acidity; salt %; fermentation time/temperature; dominant taxa; residual sugars; organic acid profile; EPS (if any); CFU/g; vegetable cultivar/source (44, 45)
Tempeh (fermented soy)	Dehulled, soaked soybeans	*Rhizopus oligosporus* (mold); cohabiting LAB/yeasts (context-dependent)	Isoflavone glycosides → aglycones (daidzein, genistein); peptides	Organic acids (lactic/acetic, minor); bioactive peptides; possible GABA (strain/process-dependent)	Bean cultivar; inoculum (*Rhizopus*) strain/CFU; fermentation time/temperature; moisture; mycelial density; isoflavone profile (aglycones/glycosides); peptide profile; CFU of cohabiting LAB/yeasts (46-48)
Miso (fermented soybean paste)	Soybeans, rice/barley koji (Aspergillus oryzae), salt	*Aspergillus oryzae* (koji); LAB and yeasts during maturation	Isoflavone aglycones; peptides; free amino acids; GABA (in some styles)	Organic acids; peptides; alcohols/esters (aroma)	Koji inoculum (*A. oryzae*) strain/enzymatic activities; salt %; maturation time/temp; pH; amino nitrogen; isoflavone and peptide profiles; viable LAB/yeast counts (49-52)
Natto (*Bacillus*-fermented soy)	Cooked soybeans	*Bacillus subtilis* var. natto	Isoflavone aglycones; menaquinones (vitamin K2)	γ-PGA; nattokinase; ammonia (minor)	B. subtilis strain; fermentation time/temp; pH; γ-PGA (g/L); nattokinase activity; isoflavone profile; CFU/g (18,22)
Kombucha (fermented tea)	Black/green tea + sucrose	*Komagataeibacter/Acetobacter spp*.; yeasts (e.g., *Saccharomyces, Brettanomyces/Dekkera*)	Remodeled tea phenolics (e.g., gallic acid release, phenolic-derived products)	Acetic, gluconic and lactic acids; bacterial cellulose pellicle (cellulosic EPS); minor ethanol	Tea type/extract (black/green; polyphenols mg GAE/L); sucrose g/L; inoculum (SCOBY) mass; fermentation time/temp; pH; titratable acidity (meq/L); residual sugars (glucose/fructose/sucrose); organic acids (HPLC);

					ethanol % v/v; cellulose pellicle mass/thickness; viable LAB/AAB/yeasts (CFU/mL) (19, 53-64)
Water kefir (tibicos)	Sucrose solution (often with dried fruits/minerals)	*Liquorilactobacillus (Lactobacillus) hilgardii*; *L. harbinensis*; *L. nagelii*; *Bifidobacterium aquikefiri*; yeasts (*Saccharomyces cerevisiae, Dekkera bruxellensis*)	Matrix-dependent phenolics from added fruits; limited direct phytochemical biotransformation vs tea/soy systems	Dextran (α-(1→6), 1→3-branched) EPS; lactic and acetic acids; CO_2; trace ethanol; glycerol; mannitol	Grain provenance (source, passage history); grain:substrate ratio (g/L); mineral content (e.g., Ca^{2+}/Mg^{2+}); fermentation time/temperature; pH; titratable acidity (as g/L lactic acid); residual sugars (glucose/fructose/sucrose); organic acids (HPLC: lactic/acetic); ethanol % v/v; mannitol (g/L) and glycerol (g/L); EPS/dextran (g/L; sol./insol.); viable LAB/yeasts (CFU/mL); volatile esters (GC–MS, optional); sensory panel (hedonics) (15, 20, 65-69, 72, 74 ,75)
Plant-milk kefir (e.g., soymilk kefir)	Soymilk (also coconut/oat/almond)	Kefir grains/cultures (LAB, AAB, yeasts) adapted to plant milks	Isoflavone glycosides → aglycones (daidzein, genistein); degradation of raffinose/stachyose	Lactic/acetic acids; EPS; enzymes (α-galactosidase/β-glucosidase activity)	Plant milk type/composition (protein, carbs, RFO); starter source (grains vs defined culture) and adaptation protocol to plant milks; inoculum dose (CFU or g/L); fermentation time/temperature; pH; titratable acidity; RFO reduction (% raffinose/stachyose); isoflavone profile (aglycones/glycosides); α-galactosidase/β-glucosidase activities; EPS (g/L); viable counts (CFU/mL); residual sugars; organic acids (HPLC); storage stability (viability, pH drift, TBARS/antioxidant capacity, if applicable) (32, 70, 71, 73, 76, 77)
Other plant matrices	Cereal and pulse doughs (e.g., wheat/rye; red bean ± wheat bran) and sourdoughs co-formulated with polyphenol-rich plant ingredients (e.g.,	LAB–yeast consortia; exemplars include *Lactobacillus/Lactiplantibacillus* K and *Kluyveromyces marxianus* in mixed-strain pulse sourdoughs; other LAB/yeast members vary by flour and process	Reductions in phytic acid (phytate), tannins, and trypsin inhibitors; increases in total phenolics/flavonoids (including gallic acid) and soluble dietary fiber; retention/enrichment of	Lactic and acetic acids (organic-acid profile linked to lower predicted/acute glycemic impact); phenolic derivatives; in vivo shifts toward higher SCFA	Flour/cultivar and co-ingredients (e.g., bran, fruit peels); inoculum (LAB/yeast species; single vs mixed strains) and dose; fermentation time/temperature and dough hydration; dough pH and titratable acidity; organic-acid profile (HPLC); phytate reduction/% and phytase activity; phenolic profile (TPC/TFC, key phenolic acids); soluble

	jabuticaba peel flour).		antioxidant compounds with fruit-peel co-ingredients (e.g., jabuticaba).	production when sourdough breads are included in diet (microbiota-mediated).	fiber; in-vitro starch digestibility (hydrolysis index/predicted GI); bread specific volume/crumb firmness and sensory; viable LAB/yeast counts (CFU) (16, 78-80)

Abbreviations: GABA, γ-aminobutyric acid; EPS, exopolysaccharide; HPLC, High-Performance Liquid Chromatography; LAB, lactic acid bacteria; CFU, colony-forming unit; γ-PGA, γ-polyglutamic acid; GAE, gallic acid equivalents: SCOBY, Symbiotic Culture of Bacteria and Yeast; ABB, Acid-Base balance; GC-MS, Gas Chromatography-Mass Spectrometry; RFO, raffinose family oligosaccharides; TBARS, Thiobarbituric acid-reactive substances; SCFA, short-chain fatty acids; TPC/TFC, Total Phenolic Content/Total Flavonoid Content; GI, glycemic index.
